# Lineage mapper: A versatile cell and particle tracker

**DOI:** 10.1038/srep36984

**Published:** 2016-11-17

**Authors:** Joe Chalfoun, Michael Majurski, Alden Dima, Michael Halter, Kiran Bhadriraju, Mary Brady

**Affiliations:** 1 Information Technology Laboratory, National Institute of Standards and Technology, 100 Bureau Drive, Gaithersburg MD, 20899, USA; 2 Materials Measurement Laboratory, National Institute of Standards and Technology, 100 Bureau Drive, Gaithersburg MD, 20899, USA; 3 Physical Measurement Laboratory, National Institute of Standards and Technology, 100 Bureau Drive, Gaithersburg MD, 20899, USA; 4 Fischell Department of Bioengineering, University of Maryland at College Park, College Park, MD, 20742, USA

## Abstract

The ability to accurately track cells and particles from images is critical to many biomedical problems. To address this, we developed Lineage Mapper, an open-source tracker for time-lapse images of biological cells, colonies, and particles. Lineage Mapper tracks objects independently of the segmentation method, detects mitosis in confluence, separates cell clumps mistakenly segmented as a single cell, provides accuracy and scalability even on terabyte-sized datasets, and creates division and/or fusion lineages. Lineage Mapper has been tested and validated on multiple biological and simulated problems. The software is available in ImageJ and Matlab at isg.nist.gov.

Automated microscopy has facilitated the large scale acquisition of live cell image data^1^ to monitor migration, morphology, and lineage tracing of large numbers of single cells or colonies in culture. However, obtaining useful quantitative dynamic data related to cell or colony behavior (including the identification of cell growth, mitosis, migration, proliferation, death, fusion, and differentiation) requires image analysis methods that can accurately segment and track cells in the presence of frequent cell-cell contacts.

A typical workflow to quantify single cell dynamics begins with segmentation, followed by tracking the segmented masks, and finally extracting dynamic tracking outputs, from which all post-processing analysis can be derived. Segmentation, the process of outlining objects of interest in digital images, is a very challenging aspect of image analysis and is usually custom-designed for a specific cell line and imaging modality. The related problem of tracking individual objects in time-series image data is also similarly constrained. Most common cell tracking techniques^2^,^3^,^4^,^5^,^6^,^7^,^8^,^9^,^10^,^11^,^12^,^13^ are linked to a particular segmentation method, where there is inherent feedback between the segmentation and the tracking algorithms (Supplementary Table 1), thus making them impractical for use across a broad range of applications. In most available tracking techniques, a segmentation method cannot easily be replaced with another more accurate one for the researcher’s specific application.

There is a need to develop tracking tools with sufficient functionality and flexibility to render themselves broadly applicable within multiple scenarios. Characteristics of an algorithm that are common to most cell biology problems^14^,^15^,^16^,^17^ include the accuracy of tracking over a range of cell contact levels (from well-separated to confluent cultures), scalability, simple communication with any segmentation method, and the minimization of non-intuitive parameters that map to the underlying mathematical models.

We developed Lineage Mapper (LM) to address these challenges (Supplementary Table 2, and Supplementary Note 1). LM detects 2D dynamic single cell behaviors: migration, mitosis, cell death, cells within sheets, and cells moving with high cell-cell contact. While Lineage Mapper was mainly developed for cell biology, it has also been successfully applied on particle tracking as we demonstrate in the validation/results section. It has six equally important and unique capabilities: **1)** LM operates on segmented masks, therefore the system is not dependent upon a particular segmentation method. In fact, LM is totally segmentation-independent: connecting segmentation results to the tracker does not require any change in the pipeline or any special input. The user has the choice of any segmentation technique including manually drawn masks as input to LM. The tool takes labeled segmented masks as input and outputs a cell lineage tree and a set of new labeled masks where each cell is assigned a unique global tracking number. **2)** In addition to the overlap information, LM uses biological properties measured from the segmented images to detect mitosis. These properties include mother cell roundness, mother cell size, daughter size similarity, and daughter aspect ratio similarity. **3)** LM uses the overlap information between current and past frames to identify and separate cells mistakenly segmented as a single cell when cell-cell contact occurs. **4)** Its execution is fast enough for real-time tracking and manages memory efficiently for large datasets. **5)** LM creates fusion lineages by tracking colony or cell merges. **6)** LM relies on a small number of biologically-derived adjustable parameters to achieve high accuracy tracking.

Figure 1 highlights LM algorithm description. The core of the LM tracking algorithm consists of four main modules. LM computes a cost function between cells from consecutive frames, detects cell collisions and separates cells by modifying input images, performs mitosis event detection, assigns tracks between cells, and finally creates the tracking outputs. The tracking outputs are: the globally labeled masks where each cell or colony is assigned with a unique label across the entire time-sequence; the mitotic lineage that shows the birth and death of each cell, the mother daughter relations, and the number of generations in a time sequence; the fusion lineage that shows the relation between cells that fused/collided together; and the confidence index. All other outputs can be derived through post-tracking processing.

## Results and Discussion

### Applicability Across Diverse Experimental Scenarios

**Figure 2:**
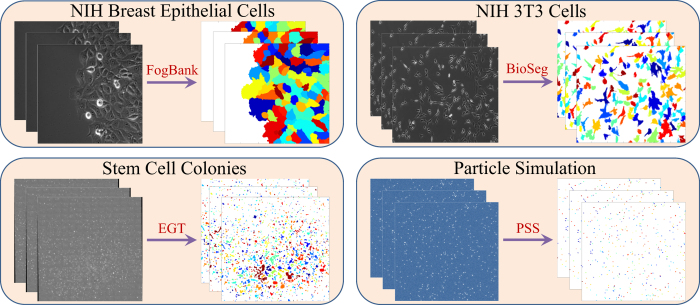
Example datasets where LM was used. Each dataset was segmented with a different segmentation method and all were tracked using LM.

Lineage Mapper has been applied on three time-lapse cell image experiments as well as on publicly available datasets of simulated particles in motion^18^ as shown in Fig. 2. Each dataset was segmented using a different segmentation method. For the first application^19^,^20^, we tracked MCF10A breast epithelial cells that are connected together and move as a sheet, a situation that is commonly encountered with epithelial cells and is of interest for understanding mechanisms of sheet-like cell migration observed during development and migration of some cancer cells^21^. The sheet of cells was segmented using the custom built segmentation technique FogBank^22^. For the second application^23^, we tracked the movement of NIH 3T3 cells to examine the dynamic regulation of tenascin-C promoter activity. Tenascin-C protein plays a critical role in development, wound healing, and cancer progression. NIH 3T3 is a fibroblast-like cell line where there is predominantly single cell migration with frequent shape changes and collisions between moving cells. This dataset was segmented using the custom built segmentation BioSeg^24^. For the third application^25^, we tracked colonies of pluripotent stem cells, in which colonies move, grow, and fuse (merge) with other colonies. Pluripotent stem cells exist in a privileged developmental state with the potential to form any of the cell types of the adult body. These cells grow as isolated colonies, with each colony consisting of tens to thousands of cells. There is an interest in tracking these colonies to understand the temporal relation between gene expression and cell state, in order to potentially engineer the cell state for regenerative medicine applications^25^. Since an individual colony is larger than the size of a single camera frame, colony tracking can only be done from time-lapse stitched image mosaics. This dataset of stem cell colonies is segmented using EGT^26^. For more detailed information about the tracking challenges, our corresponding segmentation solutions, and the biological discussions for each of the four problems, please refer to Supplementary Note 2.

**Figure 3:**
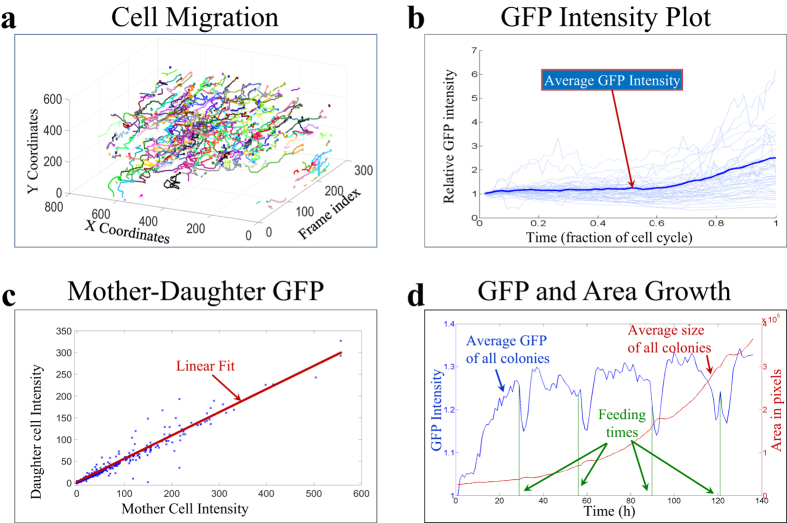
Examples of post-processing tracking outputs to analyze biological hypotheses.

The Lineage Mapper tracking outputs can be post-processed to derive additional information. Figure 3 displays some of the post-processing analysis performed for the three previously mentioned biological problems. Figure 3a displays the migration rate of cells within the MCF10A breast epithelial sheet. Figure 3b shows that individual cells vary substantially in their expression patterns over the cell cycle, but that on average TN-C promoter activity increases approximately 60% through the cell cycle. Figure 3c shows the mother-daughter GFP plot over 4 generations of cells showing an approximately even division of GFP between the two 2 daughter cells. Figure 3d shows the changes in average human embryonic stem cell colony GFP and its correlation with the 24 h feeding schedule required by these cells. Figure 3d also shows the change in aggregate colony area of all the colonies tracked over the 5-day period, thus allowing tracking of the health of the stem cell preparation by monitoring colony growth rate.

### Qualitative Accuracy Measurement

Before performing a quantitative assessment of LM performance, we first assessed its performance visually or semi-quantitatively on all the datasets covering three biological applications, described below.

#### Dataset 1

Three replicas of MCF10A breast epithelial cell sheets^19^ are imaged on different days to test LM tracking performance. Each replica is comprised of 4 wells imaged in phase contrast modality using 10x objective every 2 minutes for 2 h generating a total of 60 images per well. The total number of images in that experiment is 3 replicas × 4 wells×60 time points = 720 images. The tracking performance was validated visually by looking at the movies and checking the mitosis lineage output.

#### Dataset 2

A 6-well plate was used for the TN-C project where 2 Field of Views (FOV) were manually located per well for a total of 12 FOV per plate. Each FOV was imaged every 15 minutes for more than 62 h using a 10x (0.3 NA) objective. This experiment was duplicated twice on different dates for a total of 36 FOV with a total of 250 frames per FOV.

This dataset is too large to visually verify the tracking performance on all 36 FOV. Hence, a small subset of all cells present in the 36 FOV were used for comparison. These cells of interest, division-to-division cells, are chosen based on the fact they remained in the field of view throughout a complete cell cycle, and that they were well separated from other cells in the FOV. The quality of the automated segmentation and LM tracking is assessed by comparing the similarity of the computed biological outputs derived from the automated method with the manually identified cells of interest. There was 257 manually detected, segmented and tracked cells over the 36 FOV whereas 344 cells were automatically detected. We found a high similarity between the outputs generated from the automated segmentation and tracking and those generated from the manual ones^23^ (Supplementary Note 3), suggesting that LM can accurately track division-to-division processes and measure cell proliferation times.

#### Dataset 3

This dataset contains 161 time points of 18 × 22 individual camera frames of human embryonic stem cells with a 10% overlap between frames in both the X and Y directions. Images were collected through time in the form of contiguous stitched mosaics in phase contrast and GFP image modalities. Each mosaic image size is around 22000 × 22000 pixels (≈1 GB) for a total movie size of ≈320 GB. Three replicates were created on different dates for a total ≈1 TB of image data. LM tracked these colonies which move, grow, and merge with other colonies^25^. The tracking performance was validated visually by looking at the movies and checking the fusion lineage for any mistakes. This testing was done by four independent observers over the entire dataset using an online Web Image Processing system^27^ at isg.nist.gov.

The stem cell colony datasets presented additional challenges from a scalability perspective (execution speed, and memory management), and colony merging perspective: Tracking colony identity is the inverse of tracking single cells as colonies change their identity through time not by division but rather by fusion (merging), producing a reversed lineage tree.

### Quantitative Accuracy measurement

After the qualitative assessment, we used 3 datasets with ground truth tracking, to quantitatively assess LM tracking performance: One simulated dataset^18^ and two real, manually segmented and tracked datasets.

#### Simulated Datasets

We used a publicly available simulated dataset of particles in motion^18^. This dataset includes three particle densities (low, medium, and high) for 100 time lapse images of 4 particle motion scenarios: vesicles, microtubules, receptor, and viruses for a total of 12 simulated datasets. It is segmented using a custom-made Particle Simulation Segmentation (PSS) (Supplementary Note 3).

**Figure 4:**
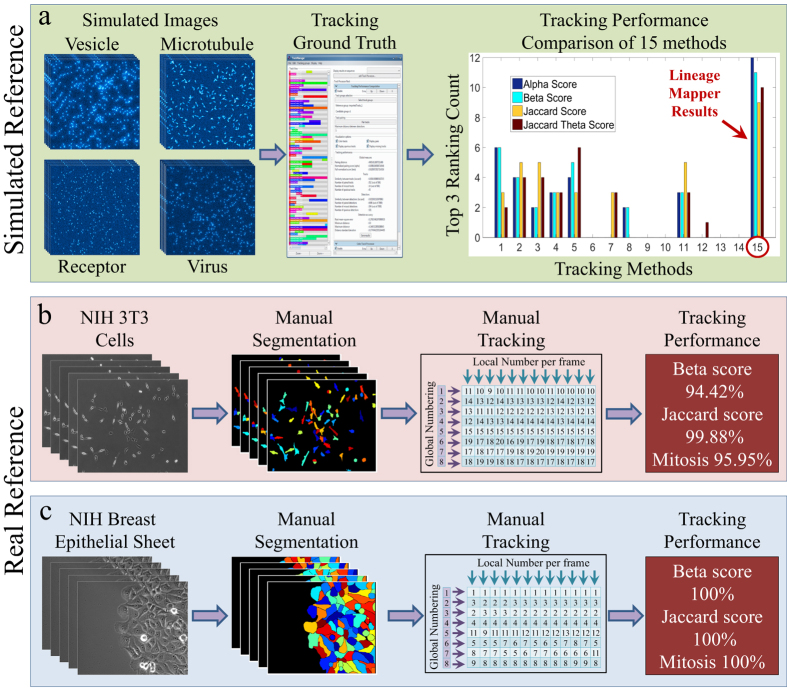
Cell and particle tracking performance of Lineage Mapper on real and simulated datasets. (**a**) Tracking accuracy measured on 12 simulated reference datasets for performance quantification and comparison between 15 trackers. (**b,c**) Tracking accuracy measured on manual segmentation and tracking of two randomly chosen time-lapse images of NIH 3T3 fibroblast cells and MCF10A breast epithelial sheets respectively.

LM tracking performance is compared against 14 existing particle tracking tools (Supplementary Table 3) on the simulated datasets (Fig. 4a). The accuracy of any tracking method was computed using four metrics: Alpha, Beta, Jaccard, and Jaccard Theta^18^. The summary of the accuracy results is reported by the number of times a particular method ranked among the top 3 positions for a given scoring metric across all 12 datasets. This analysis measures the robustness of a tracking technique across multiple particle motion scenarios and scoring metrics. LM performed well amongst the tested particle tracking methods^18^ (Fig. 4a) and scored in the top three: 12/12 with the Alpha score, 11/12 with the Beta score, 9/12 with the Jaccard score, and 10/12 with the Jaccard Theta score (Supplementary Note 3).

The simulated dataset is used as a fair example to compare the LM algorithm to existing trackers. The challenge gave participants a training set to adjust their corresponding segmentation and tracking algorithm parameters and a test set where the results can only be computed by the provided tool (a plugin to Icy^28^). Hence, all trackers had their parameters tuned by the algorithm designers who submitted their best results to the tracking challenge. This evaluation methodology eliminates the knowledge discrepancy between users of the algorithm and the designers of the algorithm. Just like the other participants in the tracking challenge, we adjusted the parameters of our tracker on the training data, just as if we were a delayed participant in the tracking challenge. Even though LM was mainly motivated and designed for cell biology, it can also be applied to particle tracking problems and still perform competitively.

#### Real Datasets

One randomly chosen MCF10A breast epithelial cell sheets time-sequence (59 frames, 5722 tracking decisions, and 6 mitosis events and One randomly chosen NIH 3T3 fibroblast experiment (238 frames, 8091 tracking decisions, and 148 mitosis events) were manually segmented and tracked by expert scientists (Supplementary Note 3). Both manual segmentations and tracking data were inspected by a second expert to reduce human errors. LM tracking and mitosis detection accuracies measured between 94.42% and 100% (Fig. 4b,c) on these biological datasets.

In summary, we presented a new 2D cell tracking system called Lineage Mapper for live cell image analysis and particle motion tracking. Its utility is demonstrated on applications where cell tracking is difficult because cells are in contact, change shape, grow, divide, or merge over multiple generations. This software, available both in ImageJ and MATLAB at isg.nist.gov, is easy to use through a graphical user interface, and is completely independent from any cell segmentation method.

## Methods

### Cost Function

Tracking is performed by assigning a cost between cells from the previous frame to cells in the current frame^29^. The cost function consists of the sum of three weighted terms computed between cells at consecutive time points: the amount of overlap, the centroid distance, and the size change. The weights are provided for flexibility and allow the basic algorithm to be tailored for use with different cell lines and image acquisition conditions (simulated dataset in Supplementary Note 3). This cost function carries desirable properties such as the ease of including additional tracking criteria by simply adding new terms (like shape metrics, or texture derived metrics, etc.…).

The cost function used for tracking decisions between time t and t + 1 is:


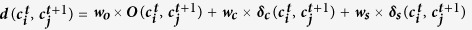


where: *w*_*o*_ is the weight of the overlap term, *O* is the overlap between area of a cell at time t and another at time t + 1, *w*_*c*_ is the weight of the centroid offset term, *δ*_*c*_ is the distance metric between cell centroid at time t and another at time t + 1, *w*_*s*_ is the weight of the cell size term, *δ*_*s*_ is the distance metric between cell size at time t and another at time t + 1. *c*^*t*^_*i*_ is cell i at time t and 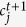 is cell *j* at time t + 1. Robustness analysis of these parameters is presented in Supplementary Note 3.

### Collision Detection

Cell collision is a term we use to describe a group of cells that are correctly detected as individual cells at time *t,* but mistakenly segmented as a single cell cluster at time *t* + *1*, when they migrate very close to one another (Supplementary Note 1).

Even for very accurate segmentation techniques, adjacent groups of cells can still be mistakenly considered as a single cell. In order to correctly keep track of their motion, a feedback loop from the tracking information to the segmented masks is implemented to separate incorrectly grouped cells into multiple single cell segments. This option can be disabled by the user to allow object merging or fusion, for example, when tracking cell colonies. When the user enables cell fusion, LM builds a fusion tree where multiple tree branches merge together to form one single branch.

### Mitosis Detection

For accurate mitosis detection, LM relies on four general biological indicators that describe mitosis events across most cell lines: 1) Mother cells divide normally into two daughter cells via mitosis. 2) Before that event the mother cell shape becomes more circular. 3) During the process the mother cell has a very low migration rate which results in a significant overlapping area with both daughter cells. 4) Daughter cells have similar size and shape (Supplementary Note 1). The Lineage Mapper implements all of the above models in the algorithm as user adjustable parameters to detect correct mitotic events for a particular cell line.

### Track Assignment

After handling mitosis and cell collision, a track (when possible) will be assigned between the remaining untracked cells. Tracks are assigned such that a cell A at time t can share a track with only one cell B at time t + 1 and vice versa. The unassigned cells at time t are considered as either leaving the Field of View (FOV) through the borders or mitotic mothers. The unassigned cells at time t + 1 are considered as either entering the FOV from the borders or originating from mitosis. The optimal solution to this assignment problem is achieved using the Hungarian algorithm^30^,^31^, which finds an optimal solution that minimizes the sum of the above-defined tracking costs over all possible tracking assignments after handling mitosis and collision.

Once the individual cell mappings between consecutive frames have been computed, the frame-to-frame mappings are combined to produce a complete life cycle track of all the cells in the image set. Cell labels assigned by the segmentation process for each frame are replaced by unique global track labels that identify each cell over time across the entire set of images. The resulting tracked mask is saved in TIFF format as part of the output of the Lineage Mapper. For each tracked cell a confidence index (Supplementary Note 1) is computed which represents the confidence in that cells tracking through its entire cell cycle.

### Tracking Output

For the Lineage Mapper there are four properties that completely describe the tracking output: 1) the globally labeled masks where each cell or colony is assigned a unique label for the entire time-sequence, 2) the cell lineage that shows the cell birth, cell death, the mother daughter relations, and the number of generations in an image set, 3) the division and/or fusion lineage that shows the relation between cells that divided and/or collided, and 4) the confidence index. All other output can be derived from these canonical ones. For instance, from the tracked masks, one can compute the location of each cell centroid and plot the respective migration rate of each cell. All geometric features like circularity, aspect ratio, etc. can be derived from these masks. All intensity features of each cell like the average GFP intensity, texture features etc. can be derived from the tracked masks and the GFP masks.

### Tracking performance metrics

There are two methods to map automatically generated tracks to reference tracks:

1. A cell identified by an automated algorithm will be mapped to a cell identified manually if their respective centroid distance is less than a tolerance distance *ε* (ε = 5 pixels in the simulated dataset)^18^. This method is used on the simulated datasets.
2. A cell identified by an automated algorithm will be mapped to a cell identified manually if both have maximum overlap with each other over the set of all possible partner cells. Due to the fact that centroid locations can be computed in many different ways (the geometric centroid, the weighted centroid, the weighted centroid along the principal axis etc.). This method is used on the bio-datasets.

Tracking accuracy is measured by four metrics^18^: Alpha, Beta, Jaccard and Jaccard Theta. The first three metrics are computed on the tracking decisions between consecutive frames over the image sequence, while the fourth metric is computed on the complete cell cycle. The range of each metric is [0, 1], where 0 is the worst case with all tracks being missed, and 1 is the best case where all tracks are perfectly detected. False Positive (FP) tracks exist in automated tracking but not in reference tracking. False Negative (FN) tracks exist in reference tracking but not in automated tracking. True Positive (TP) tracks are common tracks between automated and reference tracking.

1. The Alpha metric is proportional to the centroid distances between the automated tracks and the reference ones but ignores the FP tracks.
2. The Beta metric is similar to Alpha but penalizes for FP tracks.
3. The Jaccard metric is computed as the following ratio: 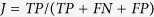.
4. The Jaccard Theta metric has the same formula as Jaccard but is applied on the complete cell cycle instead of consecutive tracking decisions.

Sources of experimental data:

#### NIH 3T3 fibroblast cells

NIH-3T3 mouse fibroblasts (ATCC, Manassas, VA) were seeded into a 6-well plate at a cell concentration of 1200 cm^2^ for each time lapse imaging experiment. This study was approved by NIST and carried out in accordance with the approved guidelines. A complete description of the set up used to acquire this dataset was published in Halter^23^, and briefly described below. Optical microscopy images were acquired every 15 minutes for 62 h using a 10x (0.3 NA) objective on Zeiss 200 M inverted microscope equipped with an automated stage, a collimated blue LED (470 nm) fluorescence excitation source and a CoolSNAP HQ CCD camera. A 0.63x demagnifying lens was positioned in front of the CCD. Cells were maintained throughout the experiment in a humidified 5% CO_2_ balanced-air atmosphere at 37 °C using a microscope incubation chamber. Before the start of each experiment, 12 stage locations were manually identified and the *x, y*, and *z* positions were stored within the acquisition software. Immediately before the time lapse acquisition started, each field was exposed to the fluorescence excitation for 30 s to reduce background fluorescence from the growth media. At each position the image acquisition software controlled the following operations sequentially: 1) the transmitted light shutter was opened, 2) auto-focusing was performed on the phase contrast image, 3) a phase contrast image was acquired, 4) the transmitted light shutter was closed, 5) the fluorescence illumination shutter was opened, 6) a GFP image was acquired, then 7) the fluorescence illumination shutter was closed. GFP fluorescence was collected with a standard GFP filter cube set in the optical train. The CCD acquired images using 2 × 2 binning for an exposure time of 0.05 s (for phase contrast) and 0.3 s (for GFP fluorescence). All live cell images were acquired in the presence of phenol red free media. Three time-lapse image sets were generated, each containing 3 well-to-well replicates with 238 time-lapse images at 520 × 696 pixels each. A spatial calibration target was used to determine that each pixel is equivalent to an area of 3.78 μm^2^.

#### Stem cells

The H9 human embryonic stem cell line (WiCell, Madison, WI)^32^ was genetically engineered to express EGFP under the influence of the Oct4 promoter by homologous recombination using a plasmid developed in the laboratory of James Thomson and obtained from Addgene (Cambridge, MA)^33^. This study was approved by NIST and carried out in accordance with the approved guidelines of the NIST Human Subjects Protection Office. A complete description of the set up used to acquire this dataset was published in Bhadriraju^25^, and briefly described below. Stem cell cultures were maintained at 37 °C and 5% CO_2_ with 80% humidity, in a custom built incubation chamber attached to a Zeiss 200 M microscope with a Zeiss 10x, 0.3 NA objective and an automated stage. Stage, filters, and shutters were controlled by the Zeiss Axiovision software. Every 45 minutes each field of view was exposed to light from a halogen lamp for phase contrast imaging, followed by excitation light for GFP fluorescence imaging. Imaging was performed for 120 h. Because of the large size of colonies (hundreds of micrometers in diameter after several days) and to assure adequate sampling of colonies, we imaged a large contiguous area of the culture plate well. For each of the tree replicates, the stage was programmed to move from field to field with an overlap of 10% with adjacent fields for a grid area of 396 FOV (18 × 22). A spatial calibration target was used to determine that each pixel is equivalent to an area of 0.394 μm^2^. This large dataset demonstrates LM scalability. It consists of 161 time-lapse images at roughly 22 000 × 22 000 pixels for each stitched image.

## Implementation

Lineage Mapper was designed and prototyped in MATLAB before being ported to Java as an ImageJ plugin. MATLAB was selected as the prototyping language due to its extensive collection of library functions and the speed with which prototyping can be accomplished. ImageJ/Fiji was selected because of its popularity for image processing and analysis platforms for biology. Both versions have a GUI interface.

Tracking the NIH 3T3 image sequence (comprised of 238 frames, at 520 × 696 pixels per frame, with 589 cells, and 7356 tracking decisions) requires approximately 30 seconds of runtime in MATLAB and less than 2 seconds using the ImageJ plugin. Tracking the stem cell colonies (comprised of 161 frames, at 22 000 × 22 000 pixels per frame, with 1060 cells, and 32 722 tracking decisions) requires 3 h 15 minutes using the MATLAB version and 1 hour and 7 minutes with the ImageJ plugin.

Both versions require approximately the same amount of system memory (RAM) to run. Memory is required to hold two images (the previous time point and the current time point) with a few small ancillary data structures containing the tracking data. Therefore, the memory required to perform tracking scales primarily with the size of the images being tracked. Both versions are single threaded, sequentially tracking each image in the sequence.

All runtimes discussed here were generated using a 64 bit Windows 7 system with an Intel Xeon E5-2620 @2.0 G Hz with 64 GB RAM and a 7200 rpm (revolutions per minute) hard disk.

The Lineage Mapper implementation is open source and free to download, use, or modify as the user sees fit. Incorporating this tool in an image processing pipeline can be achieved via its batch (command line) mode which does not use the GUI. Both the MATLAB executable and ImageJ plugin are available from isg.nist.gov.

## Additional Information

**How to cite this article**: Chalfoun, J. *et al*. Lineage mapper: A versatile cell and particle tracker. *Sci. Rep.* **6**, 36984; doi: 10.1038/srep36984 (2016).

**Publisher’s note**: Springer Nature remains neutral with regard to jurisdictional claims in published maps and institutional affiliations.

## Supplementary Material

Supplementary Information

## Figures and Tables

**Figure 1:**
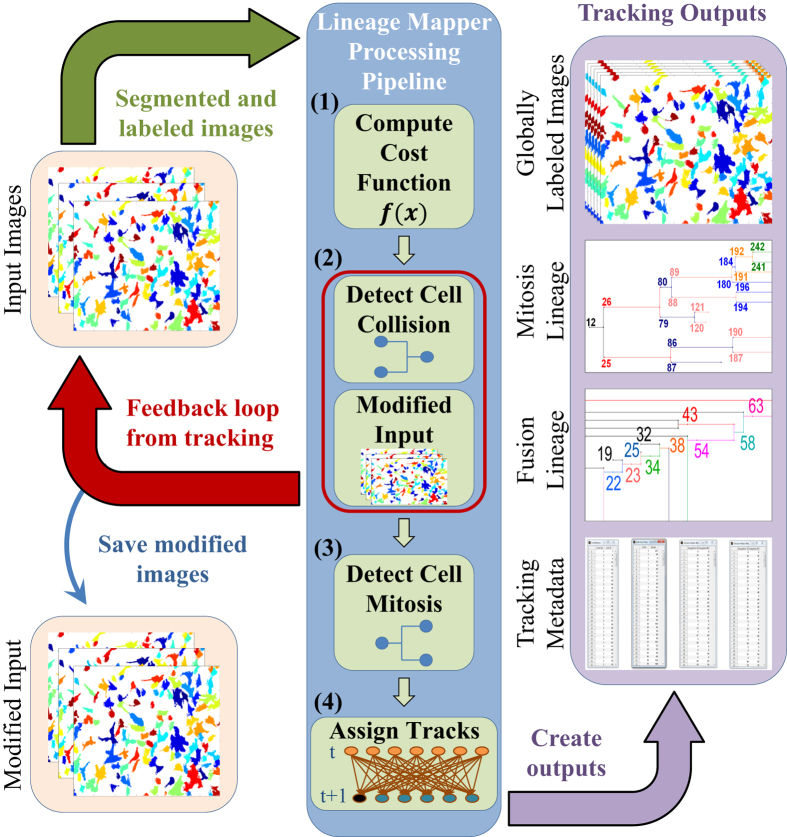
Schematic description of Lineage Mapper algorithm and output summary data and visualizations.
