# Supplementary material for: Lineage mapper: A versatile cell and particle tracker
Source: Sci Rep. 2016 Nov 17;6:36984. doi: 10.1038/srep36984 (PMC5113068; doi:10.1038/srep36984)
Supplement: Supplementary Information [file srep36984-s1.pdf]

Lineage mapper: A versatile cell and particle tracker – Supplementary Table 1 - Summary of Common Tracking Tools

Joe Chalfoun[1], Michael Majurski[1], Alden Dima[1], Michael Halter[2], Kiran Bhadriraju[3], and Mary Brady[1]

Table 1- Summary of common tracking tools compared with our Lineage Mapper

Tracking Techniques	Lever [1]	CMU Tracker [2]	Imaris [3]	BioImageXD [4]	ImageJ (Mtrack2) [5]	Lineage Mapper
Total separation from segmentation	N	N	N	N	Y	Y
Mitosis detection	Y	Y	Y	Y	N	Y
Automatic detection of cell-cell contact	N	Y	N	N	N	Y
Tracking confidence index	N	N	N	N	N	Y

Total separation from segmentation means that connecting segmentation results to the tracker does not require any change in the pipeline or any special input.

References

[1] M. Winter, E. Wait, B. Roysam, S. K. Goderie, R. A. N. Ali, E. Kokovay, S. Temple, and A. R. Cohen, "Vertebrate neural stem cell segmentation, tracking and lineaging with validation and editing.," *Nat. Protoc.*, vol. 6, no. 12, pp. 1942–52, Dec. 2011.

[2] S. Huh, "Toward an Automated System for the Analysis of Cell Behavior : Cellular Event Detection and Cell Tracking in Time-lapse Live Cell Microscopy Seungil Huh," CMU, 2013.

[3] U. Krzic, S. Gunther, and T. Saunders, "Multiview light-sheet microscope for rapid in toto imaging," *Nat. …*, vol. 9, no. 7, 2012.

[4] P. Kankaanpää, L. Paavolainen, S. Tiitta, M. Karjalainen, J. Päivärinne, J. Nieminen, V. Marjomäki, J. Heino, and D. J. White, "BioImageXD: an open, general-purpose and high-throughput image-processing platform.," *Nat. Methods*, vol. 9, no. 7, pp. 683–9, Jul. 2012.

[5] J. Schindelin, I. Arganda-Carreras, E. Frise, V. Kaynig, M. Longair, T. Pietzsch, S. Preibisch, C. Rueden, S. Saalfeld, B. Schmid, J.-Y. Tinevez, D. J. White, V. Hartenstein, K. Eliceiri, P. Tomancak, and A. Cardona, "Fiji: an open-source platform for biological-image analysis.," *Nat. Methods*, vol. 9, no. 7, pp. 676–82, Jul. 2012.

[1] Information Technology Laboratory, National Institute of Standards and Technology
2 Materials Measurement Laboratory, National Institute of Standards and Technology
3 Fischell Department of Bioengineering, University of Maryland at College Park

Lineage mapper: A versatile cell and particle tracker – Supplementary Table 2 - Characteristics of Lineage Mapper

Joe Chalfoun[2], Michael Majurski[1], Alden Dima[1], Michael Halter[2], Kiran Bhadriraju[3], and Mary Brady[1]

Table2: The critical characteristics of Lineage Mapper that allow it to be a generally applicable solution for cell tracking

**Accuracy and robustness**	An accuracy between 94.2 % and 100 % is achieved on 2 manually tracked datasets. Robustness against the choice of parameters was also shown on these 2 reference datasets. No training set is needed.
**Functional modularity**	Decoupled segmentation and tracking steps. Executes on labeled segmented images only. Simple communication to any type of segmentation.
**Scalability**	Can be applied to Big Data (TB sized) image sets with low memory footprint. For example, tracking a set of 22 000 x 22 000 pixel images through 161 time points in 1 h on a regular machine with less than 8GB of memory.
**Mitosis detection**	Overlap-based mitosis detection that uses the mother and daughter information such as roundness and size of mother cell, and aspect ratio and size similarity of daughter cells.
**Cell collision detection**	Detection of cell collision/fusion based on cell overlap. Separation of multi-cell area into multiple single cell segments. A fusion lineage plot can be produced if cell or colony merging is allowed.
**Number of input parameters**	Small number of biologically driven parameters.
**Versatility**	Successfully applied to a wide variety of applications with high accuracy achieved in each case.
**Execution speed**	Real-time tracking during acquisition is possible. It can be used in a batch mode and called from an ImageJ script. Tracking consecutive images of size 520 x 696 pixels takes 0.0125 s per frame on a regular 2.2 GHz intel i5 machine with 8GB of memory.
**Availability**	MATLAB source code and executable, and an ImageJ plugin with online help are available for downloading from https://isg.nist.gov.

[2] Information Technology Laboratory, National Institute of Standards and Technology
2 Materials Measurement Laboratory, National Institute of Standards and Technology
3 Fischell Department of Bioengineering, University of Maryland at College Park

Lineage mapper: A versatile cell and particle tracker – Supplementary Table 3 – Particle Trackers Summary

SUMMARY OF PARTICLE TRACKING TOOLS				
METHOD 1	Authors: Ivo F. Sbalzarini, Yuanhao Gong, Janick Cardinale	Email: ivos@mpi-cbg.de	Software: http://mosaic.mpi-cbg.de/?q=downloac	Form: ImageJ plugin
METHOD 2	Authors: Craig Carthel and Stefano Coraluppi	Email: stefano.coraluppi@compunetix.co	Software: Contact the authors	Form: Windows executable
METHOD 3	Authors: Nicolas Chenouard, Fabrice de Chaumont, Jean-	Email: jcolivo@pasteur.fr	Software: Contact the authors	Form: Icy plugin
METHOD 4	Authors: Mark Winter and Andrew R. Cohen	Email: acohen@coe.drexel.edu	Software: Contact the authors	Form: Matlab script
METHOD 5	Authors: William J. Godinez and Karl Rohr	Email: k.rohr@dkfz-heidelberg.de	Software: Contact the authors	Form: Java module
METHOD 6	Author: Yannis Kalaidzidis	Email: kalaidzi@mpi-cbg.de	Software: http://motiontracking.mpi-cbg.de/	Form: Windows executable
METHOD 7	Authors: Liang Liang, James Duncan, Hongying Shen, Yingke	Email: liang.liang@yale.edu	Software: Contact the authors	Form: Matlab script
METHOD 8	Authors: Klas E. G. Magnusson, Joakim Jaldén, Helen M. Blau	Email: klasma@kth.se	Software: Contact the authors	Form: Matlab script
METHOD 9	Author: Perrine Paul-Gilloteaux	Email: perrine.paul-gilloteaux@curie.fr	Software: Contact the author	Form: ImageJ plugin
METHOD 10	Authors: Philippe Roudot, Charles Kervrann, François Waharte	Email: philippe.roudot@inria.fr	Software: Contact the authors	Form: C++ code
METHOD 11	Authors: Ihor Smal and Erik Meijering	Email: i.smal@erasmusmc.nl	Software: Contact the authors	Form: Java module
METHOD 12	Author: Jean-Yves Tinevez and Spencer L. Shorte	Email: tinevez@pasteur.fr	Software: http://fiji.sc/TrackMate	Form: ImageJ/Fiji plugin
METHOD 13	Authors: Joost Willemse, Katherine Celler, Gilles P. van Wezel	Email: jwillemse@biology.leidenuniv.nl	Software: Contact the authors	Form: ImageJ plugin and Matlab script
METHOD 14	Authors: Han-Wei Dan and Yuh-Show Tsai	Email: dnadann@gmail.com	Software: Contact the authors	Form: ImageJ plugin
This table is extracted from the following publication: Chenouard, N. et al. Objective comparison of particle tracking methods. Nat. Methods 11, 281–9 (2014)				

Lineage mapper: A versatile cell and particle tracker – Supplementary Note 1 – Mitosis and Fusion

Joe Chalfoun[3], Michael Majurski[1], Alden Dima[1], Michael Halter[2], Kiran Bhadriraju[3], and Mary Brady[1]

1 Introduction

In this document we will describe in detail the mitosis detection and the fusion/collision handling cases of the Lineage Mapper. We will also describe the two kinds of lineage plotting (fusion and division) and the computation of the tracking confidence index.

[3] Information Technology Laboratory, National Institute of Standards and Technology
2 Materials Measurement Laboratory, National Institute of Standards and Technology
3 Fischell Department of Bioengineering, University of Maryland at College Park

2 Overlap-based mitosis detection

Cells eventually divide into two daughter cells by the process of mitosis. During this process, the mother cell rounds, then undergoes mitosis and cytokinesis. Figure 1 illustrates an example of a mother cell that goes into mitosis at frame *t* and divides into two daughter cells at frame *t+1*. The two images superimposed (Figure 2) reveal that the mother cell has a significant overlapping area with both daughter cells. This is due to the fact that before dividing into two daughter cells the motility of the mother cell is minimal.

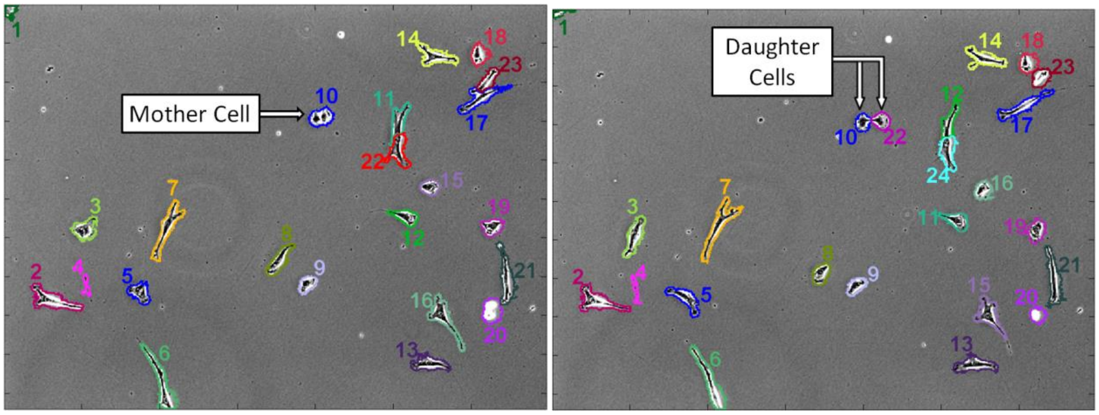

Figure 1- Example of a mitotic cell in two consecutive segmented frames overlaid on top of the original grayscale images for visualization purposes.

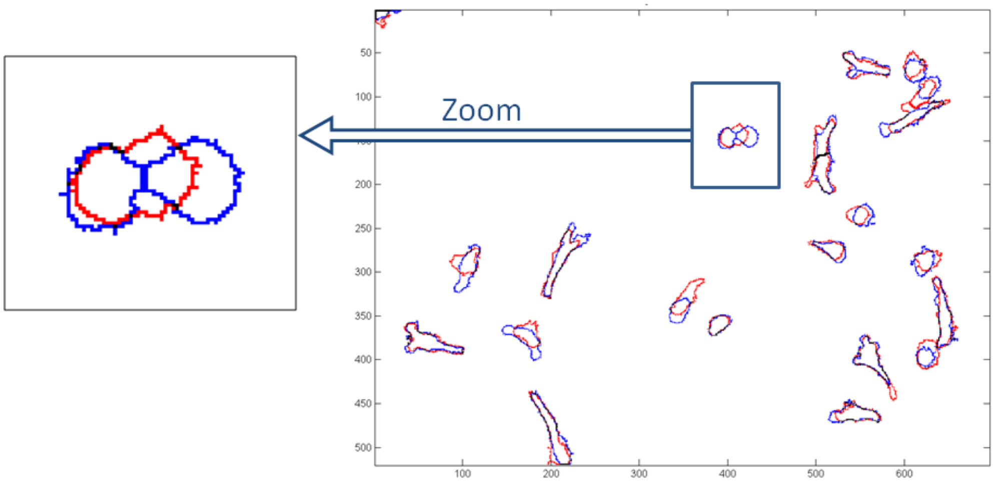

Figure 2- Superimposing image 1 (red) and image 2 (blue) and focusing on the dividing cell

The Lineage Mapper uses this cell overlap information to detect mitotic cells between two consecutive images. In general, when mitosis happens, one mother cell c_m^t from image I_t overlaps its two daughter cells c_i^{t+1} and c_j^{t+1} from image I_{t+1}. Mitosis is detected by searching the cost matrix for pairs of daughter cells at time t+1 that are tracked to the same mother cell at time t. Once these pairs of mother-daughter cells are found, the amount of overlapping between each potential daughter cell and the corresponding potential mother cell is compared against a user-defined mitosis-overlap threshold. The cell tracker will

record the mitosis event only if the amount of overlapping area of both daughter cells is above the threshold based upon their respective areas:

$$if\ \left[\left(\frac{O_{(M,D1)}}{Area_{D1}}\right) < T\right]\ or\ \left[\left(\frac{O_{(M,D2)}}{Area_{D2}}\right) < T\right]\ then\ discard\ event\quad (1)$$

Where $O_{(M,D1)}$ is the overlapping pixel area between mother cell and daughter cell 1, T is the user defined threshold for mitosis-overlap and $Area_{D1}$ is the size in pixels of daughter 1.

Figure 3 presents an example that illustrates the utility of the overlap-mitotic threshold for detecting mitosis. In that example the red outlines are cells at time t and the blue outlines are cells at time t+1. Figure 3A is a real mitotic event where each daughter cell has an overlap higher than 20% of its respective area. Figure 3B is an example of a false mitosis that can be discarded by checking the overlap of the daughter cells where clearly only one blue cell has sufficient overlap with the red cell. If cells are more mobile than usual or if the acquisition rate is low, setting this threshold to a very low value (<10 % for example) will allow Figure 3B to be considered for a potential mitosis case.

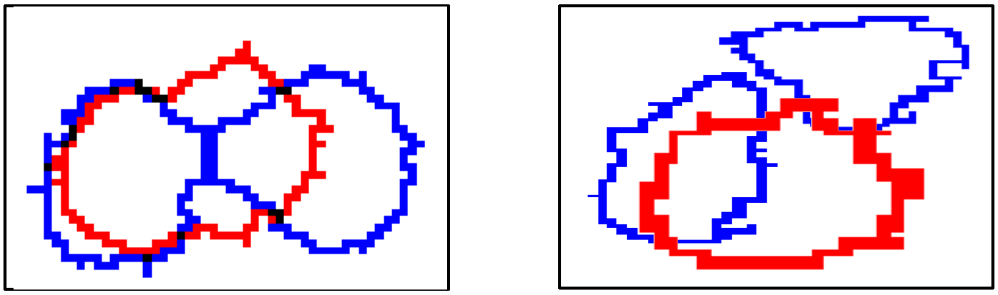

A B

Figure 3- An example illustrating the usefulness of the overlap-mitotic threshold. In both images A and B, the red outlines are cells at time t and the blue outline are cells at time t+1. Figure A is a real mitotic event where each daughter cell has an overlap higher than 20% of its respective area. Figure B is an example of a false mitosis that can be discarded by checking the overlap of the daughter cells where clearly only one blue cell overlaps sufficiently with the red cell and the other does not.

For the potential mitoses that are not discarded by the overlap threshold, three conditions need to be satisfied before declaring these events a real mitosis:

(1) Cell roundness of all potential mother cells is checked n frames before the mitosis event, where n is a user defined value. The roundness is measured by the following formula: $R = 4\pi \times a/(p)^2$ where a is the area and p the perimeter. This metric is equal to 1 for a perfect circle and decreases in value until reaching 0 for a shape similar to a line. If a potential mother cell does not meet the roundness threshold, the corresponding mitosis is discarded. To disable this ability simply select a roundness threshold of 0.

(2) Size similarity between the two daughter cells is checked against a user defined threshold and the potential mitosis is discarded if similarity is below the user-defined threshold. The similarity metric is computed by following equation 2 below where s is the similarity metric that ranges from 1 (perfect size similarity) to 0 (worse case) and s_{D1} is the size of daughter 1.

$$s = 1 - \left|\frac{s_{D1} - s_{D2}}{s_{D1} + s_{D2}}\right| \ (2)$$

(3) Aspect ratios of the two daughter cells are compared, and the potential mitosis is discarded if similarity is below the user-defined threshold. The similarity metric is computed by following equation 3 below where s is the similarity metric that ranges from 1 (perfect size similarity) to 0 (worse case) and ar_{D1} is the aspect ratio of daughter 1.

$$s = 1 - \left|\frac{ar_{D1} - ar_{D2}}{ar_{D1} + ar_{D2}}\right| \ (3)$$

3 Overlap-based cell collision/Fusion management

Cell collision is a term used to describe a group of cells that are correctly detected as individual cells at time *t*, but when they migrate at time *t*+1 they become so adjacent to each other that segmentation techniques mistakenly consider them as one single cell. Even for extremely accurate segmentation techniques, adjacent groups of cells can still be mistakenly considered as one single cell. In order to correctly segment these cells and track their motion, a feedback loop from tracking to the segmentation is created to separate the initially segmented combined cell cluster into more accurately segmented single cells. To illustrate the feedback loop, we will consider the example illustrated in Figure 4, where seven cells exist in the field of view of the phase contrast image. The corresponding segmented image reveals only three distinct cell clusters.

Cell collision can be identified between two consecutive frames, t and t+1, based on the information the cell tracker gathered from frame t. In general a collision case is when multiple cells at time t, $c_i^t, i = 1..m,\ m$ is the number of colliding cells from image I_t, are tracked to the same cell c_j^{t+1} in image I_{t+1} (Figure 4). Just like the mitotic detection case, a user defined minimum cell overlap threshold is set to filter out the bad collision cases.

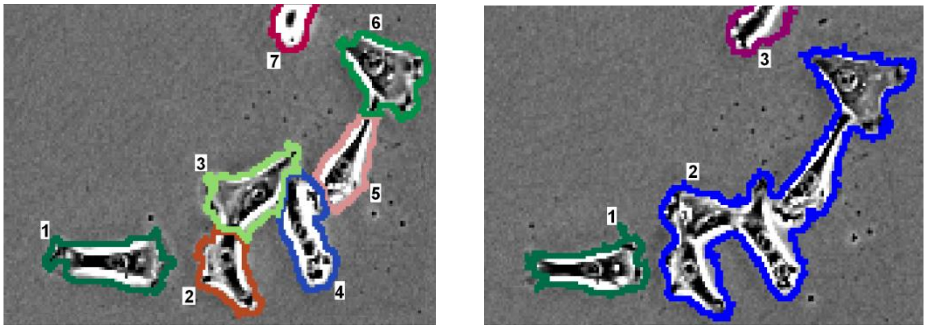

Figure 4- Tracking collision between consecutive frames t (left) and t+1 (right)

In Figure 4, Cell c_1^t is tracked to cell c_1^{t+1}. Cells $c_2^t, c_3^t, c_4^t, c_5^t$ and c_6^t are tracked to cell c_2^2 and cell c_7^t is tracked to cell c_3^{t+1}. The potential colliding region c_2^{t+1} and the colliding cells $c_2^t, c_3^t, c_4^t, c_5^t$ and c_6^t are identified. If the identified cells do not meet the collision-threshold they are eliminated from the potential collision cell list. In this example, all the colliding cells meet the threshold because they all have high overlapping area with cell c_2^{t+1}. To separate the single cell cluster into several smaller cells, the segmented masks will be modified and a new corrected segmented image is formed as shown in Figure 5. This image will be used as new input to the cell tracking algorithm and the cost matrix will also be updated accordingly. The assignment of pixels from the common cell area c_2^{t+1} is made so that pixels that

overlap across frames are assigned to the individual cell from frame *t*, and the remaining non-overlapping pixels are assigned to the closest cell neighbor.

It is very important to note that this feedback loop operates only on segmented masks and thus the separation of the group of cells into single cells may generate some cell edges that do not follow the real curvature of the cell.

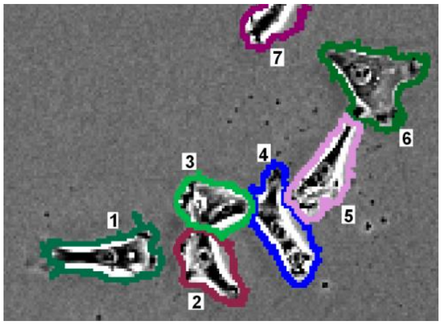

Figure 5- new input to the cell tracker at time t+1 after correcting the previously segmented mask. The cell area c_2^{t+1} is cut into 5 single cell segments.

4 Tracking assignments and output

After handling mitosis and cell collision/fusion, a track will be assigned between the remaining cells at time *t* and the remaining cells at time *t+1*, when possible. Tracks are assigned such that a cell A at time *t* can share a track with only one cell B at time *t+1* and vice versa. The unassigned cells at time *t* are considered dead (i.e. cells leaving the image through the borders, mitotic mother cells, or cells that fused together if fusion is allowed) and the unassigned cells at time *t+1* are considered newborn cells (i.e. cells entering the image from the borders, cells originating from mitosis, or cells that are born from fusion if it is allowed). In order to achieve such a solution, the Hungarian algorithm is applied on the cost matrix [1]. By using this algorithm we are able to find an optimal solution that minimizes the sum of the above-defined tracking costs over all possible tracking assignments after handling mitosis and collision/fusion.

Once the individual cell mappings between consecutive frames have been computed, the frame-to-frame mappings are combined to produce a complete life cycle track of all cells in the time-lapse image set. The sequential cell numbers that were assigned by segmentation for each frame are replaced by unique track numbers that identify the movement of each cell over time across the entire image set. Therefore a unique label or track number L_k will be associated with each uniquely identified cell, $k = 1, 2, \dots, n$ where n represents the total number of unique cells found in the image set. The pixels in the images are relabeled to reflect the new track numbers such that when a given cell is assigned with a tracking number, L_k, the pixels from all images that belong to this cell will all have the same value L_k. This is formally stated as follows.

$$if\ c_i^t \xleftrightarrow{L_k} c_j^{t+1} \Rightarrow c_i^t = L_k = c_j^{t+1}$$

$$\Rightarrow \forall(x, y)\ /\ p(x, y) \in (c_i^t\ \vee\ c_j^{t+1}), p(x, y) = L_k\ (4)$$

5 Lineage Plotting

Lineage Mapper has the ability to plot 2 kinds of lineage trees (Figure 6):

(1) The first type of plot is the regular lineage tree that shows the mitosis events and the cell cycles.

(2) The second type plots cell or colony fusion or merging. When the user checks the enable fusion checkbox, a cell fusion lineage tree is built by the Lineage Mapper that shows the fusion tree as the reverse of the division lineage tree, displaying multiple cells or colonies that fused together at a time t and created a new group of cells or colonies at time t+1.

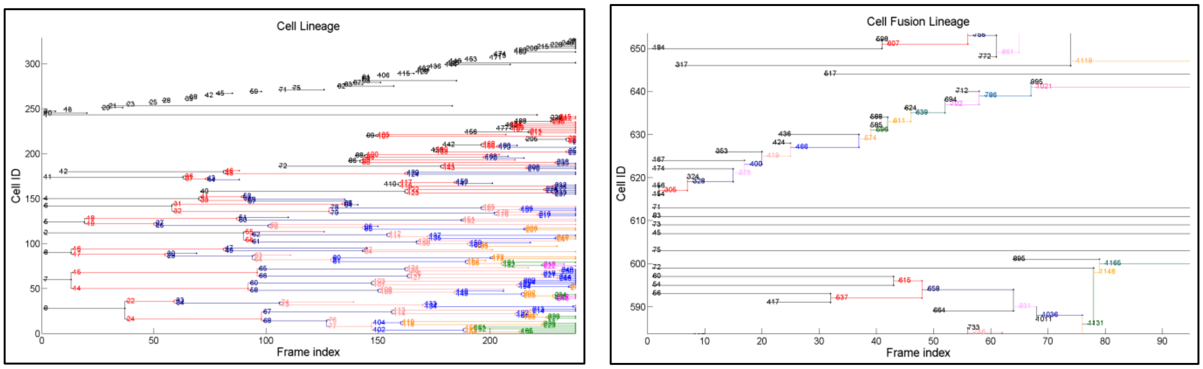

Figure 6- Lineage Plots: (left) regular lineage tree that shows mitosis and cell cycle, (right) fusion lineage that shows cell or colony fusion or merging

6 Confidence Index

The Lineage Mapper outputs a confidence index for each tracked object in the time-lapse sequence. The confidence index is an indicator of how well we trust the track of a given cell during its entire cell cycle. The computation of this index is based on user input, reflecting choices based on individual experiments. The confidence index is based on points. Each component in the equation contributes points which are added to the index. At the beginning all cells start with a confidence index of 1. Three components can affect the computation of the confidence index; each one can be disabled if needed:

$$\mathrm{CI}_c = \mathrm{L_c} + \mathrm{B_c} + \mathrm{D_c} + 1 \qquad (5)$$

Where

- CI_{cell} is the Confidence Index for a cell
- $\mathrm{L_c}$ is a binary component based on a user-defined minimum cell life cycle threshold, mc, measured in number of frames. A cell cycle is the difference in frames between the time when the cell last appeared in the field of view ($death(cell)$) and the time when it first appeared ($birth(cell)$). This component is computed as follows:

$$\mathrm{cell\_cycle(cell)} = \begin{cases} 0, if\ [death(cell) - birth(cell)] < mc \\ 1, if\ [death(cell) - birth(cell)] \geq mc \end{cases}$$

- $\mathrm{B_c}$, border cell is a binary component where a cell that is not touching the border during its entire life time will gain a point in the confidence index computation.
- $\mathrm{D_c}$, cell density is the measure of how many neighboring cells the current cell touched in its entire life time. This component is inversely proportional to the number of cells the

current one touches. The confidence index decreases when the number of touching cells increases. It is computed as follows:

$$D_c = \frac{1}{(\sum T_c + 1)}$$

Where $\sum T_c$ is the sum of the number of cells that the current cell touched during its entire life time.

7 References

[1] D. Dasgupta, G. Hernandez, D. Garrett, P. K. Vejandla, A. Kaushal, R. Yerneni, and J. Simien, "A comparison of multiobjective evolutionary algorithms with informed initialization and kuhn-munkres algorithm for the sailor assignment problem," *Proceedings of the 2008 GECCO conference companion on Genetic and evolutionary computation - GECCO '08*, p. 2129, 2008.

Lineage mapper: A versatile cell and particle tracker - – Supplementary Note 2 - Biological Applications

Joe Chalfoun[4], Michael Majurski[1], Alden Dima[1], Michael Halter[2], Kiran Bhadriraju[3], and Mary Brady[1]

In this document we present the Lineage Mapper as applied to several biological problems that demonstrate different tracking challenges.

1- LM tracked a sheet of cells that are connected together and move with the sheet, a situation that is commonly encountered with epithelial cells and is of great interest for understanding mechanisms of sheet-like cell migration observed during development and in the migration of some cancer cells [1].
2- LM tracked the movement of NIH3T3 cells, a fibroblast-like cell line where there is predominantly single cell migration with frequent shape changes and collisions between moving cells.
3- LM tracked colonies of pluripotent stem cells, in which colonies move, grow and fuse (merge) with other colonies.

The tracking challenges and our corresponding solutions are presented in the following sub-sections.

[4] Information Technology Laboratory, National Institute of Standards and Technology
2 Materials Measurement Laboratory, National Institute of Standards and Technology
3 Fischell Department of Bioengineering, University of Maryland at College Park

1 MCF10A Breast Epithelial Sheets

Many cell lines that are currently being studied for medical purposes, such as cancer cell lines, grow in confluent sheets. These cell sheets typically exhibit cell line specific biological properties such as the morphology of the sheet, protein expression, proliferation rate, and invasive/metastatic potential. However, cell sheets are comprised of cells of different phenotypes. For example, individual cells in a sheet can have diverse migration patterns, cell shapes, can express different proteins, or differentiate differently. Identifying phenotypes of individual cells is highly desirable, as it will contribute to our understanding of biological phenomena of tumor metastasis, stem cell differentiation, or cell plasticity. Time-lapse microscopy now enables the observation of cell cultures over extended time periods and at high spatiotemporal resolution. Furthermore, it is now possible not only to label cells with fluorescent markers, but also to express fluorescently labeled protein, enabling spatiotemporal analysis of protein distribution in a cell sheet at a cellular level. For more information about this project please refer to the following two publications [2][3].

To assess properties of individual cells within the observed sheet, however, it is necessary to accurately track these cells in a fully automated fashion. Segmentation of MCF10A breast epithelial sheet cells was done using the custom built segmentation FogBank [4].

The challenges of this dataset for cell tracking are: (1) high cell density, (2) continuous contact between cells, and (3) tracking mitosis (cell-division) within a contiguous cell sheet. The automated results are compared to a manually derived tracking done by an expert using only the labeled images. Accuracy of 100 % tracking was achieved including the mitotic detection of cells within the sheet.

2 NIH 3T3 Cells

Despite numerous studies, the regulation of the extracellular matrix protein tenascin-C (TN-C) remains difficult to understand. By using live cell phase contrast and fluorescence microscopy, the dynamic regulation of TN-C promoter activity is examined in an NIH 3T3 cell line stably transfected with the TN-C gene ligated to the gene sequence for destabilized Green Fluorescent Protein (GFP). By using the Lineage Mapper, we found that individual cells vary substantially in their expression patterns over the cell cycle, but that on average TN-C promoter activity increases approximately 60 % through the cell cycle. We also found that the increase in promoter activity is proportional to the activity earlier in the cell cycle. This work illustrates the application of live cell microscopy and automated image analysis of a promoter-driven GFP reporter cell line to identify subtle gene regulatory mechanisms that are difficult to uncover using population averaged measurements. The fully automated image segmentation and tracking are validated by comparison with data derived from manual segmentation and tracking of single cells. More detail about this work can be found in [5] and [6].

3 Stem Cell Colonies

Pluripotent stem cells exist in a privileged developmental state with the potential to form any of the cell types of the adult body. Hence, there is great interest in understanding the relation between gene expression and cell state, in order to potentially engineer cell state for application to regenerative medicine. We used a cell line expressing GFP under the control of a critical pluripotency related transcription factor, OCT-4, to understand how normal stem cell cultures behave during routine feeding of cultures. These cells grow as isolated colonies, each colony comprising tens to thousands of cells as the culture progresses.

Because individual colony size is larger than the size of a single camera frame, colony tracking can only be done from movies of mosaics. In our case, we made a movie of 18 x 22 individual camera frames (total mosaic size ≈1GB) with a 10% overlap between frames in both X in Y directions, over 161 time points (total movie size ≈ 350 GB). Images were collected through time in the form of contiguous mosaics in phase contrast and GFP channels.

The phase contrast images of the colonies are used to segment colonies and generate colony masks using the Empirical Gradient Threshold (EGT) technique [7]. A gradient image is formed from the original image, and the foreground and background distributions of gradient magnitude values are separated based on their overlap (Figure 7). The masks are then overlaid on the GFP image to compute GFP intensity per colony.

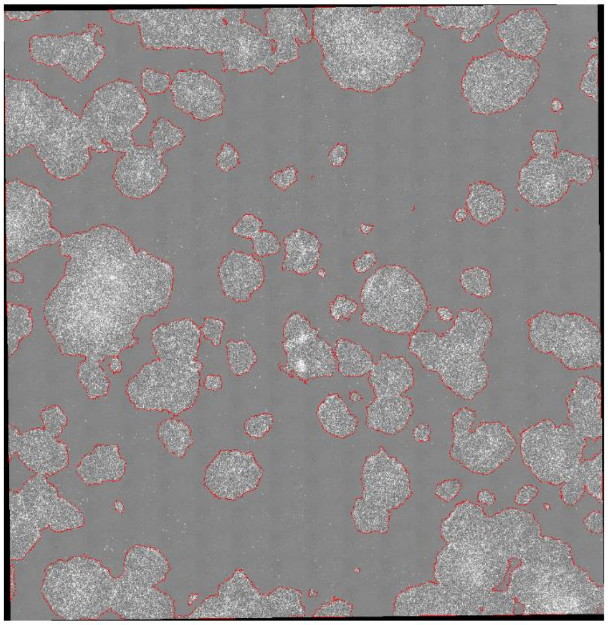

Figure 7- Segmentation of stem cell colonies overlaid on original mask.

This dataset presents additional challenges to the cell tracker: (1) scalability challenges (memory management, execution speed and accuracy in the measurement of very large data sets) that the cell tracker needs to handle properly. (2) Tracking colony identity is the inverse of tracking single cells as colonies change their identity through time not by division but rather by merging (fusion), producing a reversed lineage tree.

Maintenance of pluripotent state requires daily feeding of the cells. However, it is not known if the 24 h bolus feeding of the cells affects colony behavior. In order to observe how daily feeding affects pluripotent stem cell colonies, we tracked the colonies over a period of 5 days. We normalized the average GFP intensity of each colony by the value of the average GFP of its birth time (first appearance in the time sequence). For each time point, we compute the average normalized GFP intensity for all the colonies whose size is bigger than a user-defined threshold to filter out the noise. Figure 8 shows the plot of the average normalized GFP intensity on the left axis. The right axis displays the average area occupied by the colonies at a given time point. We observed a smaller, transient increase in colony area over the long term growth trend that lasted for 5 hours after each feeding event. This increase was consistent across all datasets. Corresponding with the changes in colony areas, we observed a dip in GFP intensity which occurred at the time of cell feeding and at a frequency of approximately 24 h, lasting for the same

amount of 5 hours after feeding. We speculate that this response may be related to the bolus addition of growth factors which are known to be depleted during culture. Our cultures were maintained in E8 media, which has the growth factors basic fibroblast derived growth factor (bFGF), transforming growth factor (TGF□), and insulin, all of which can potentially induce signaling that alters actin polymerization and alter cell morphology. While such responses have been studied at great depth in somatic cells such as fibroblast, there is little to know information on how sensitive the motile behavior of pluripotent stem cell is to growth. It would be interesting to directly examine in future studies if the feeding related morphological dynamics are indeed due to changes in actin cytoskeleton organization, and if one or more of the growth factors play a dominant role in this response.

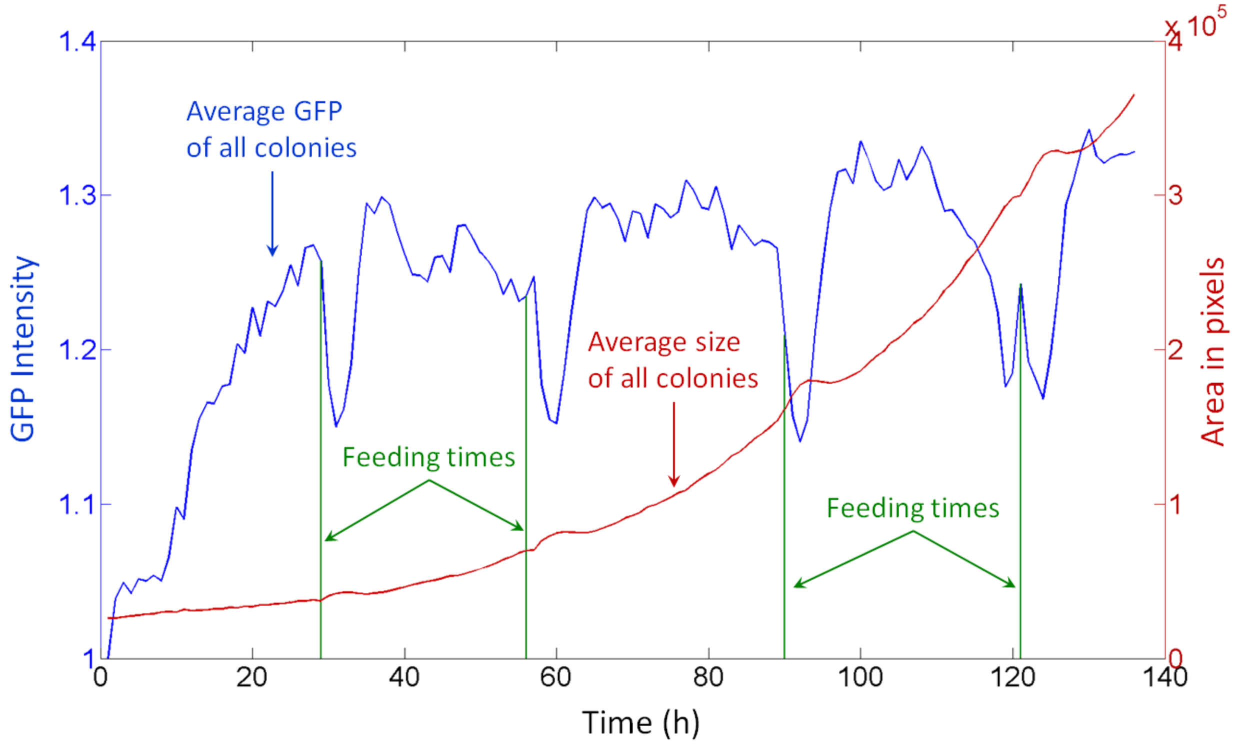

Figure 8- Example of average GFP intensity and size of all colonies with feeding time overlaid.

4 References

[1] J. H. Kim, X. Serra-Picamal, D. T. Tambe, E. H. Zhou, C. Y. Park, M. Sadati, J.-A. Park, R. Krishnan, B. Gweon, E. Millet, J. P. Butler, X. Trepat, and J. J. Fredberg, "Propulsion and navigation within the advancing monolayer sheet.," *Nat. Mater.*, vol. 12, no. 9, pp. 856–63, Sep. 2013.

[2] M. C. Weiger, V. Vedham, C. H. Stuelten, K. Shou, M. Herrera, M. Sato, W. Losert, and C. a Parent, "Real-time motion analysis reveals cell directionality as an indicator of breast cancer progression.," *PLoS One*, vol. 8, no. 3, p. e58859, Jan. 2013.

[3] C. H. Stuelten, J. I. Busch, B. Tang, K. C. Flanders, A. Oshima, E. Sutton, T. S. Karpova, A. B. Roberts, L. M. Wakefield, and J. E. Niederhuber, "Transient tumor-fibroblast interactions increase tumor cell malignancy by a TGF-Beta mediated mechanism in a mouse xenograft model of breast cancer.," *PloS one*, vol. 5, no. 3. p. e9832, Jan-2010.

[4] J. Chalfoun, M. Majurski, A. Dima, C. Stuelten, and A. Peskin, "FogBank : A Single Cell

Segmentation across Multiple Cell Lines and Image Modalities," *BMC Bioinformatics*, vol. 15, no. 431, 2014.

[5] M. Halter, D. R. Sisan, J. Chalfoun, B. L. Stottrup, A. Cardone, A. a. Dima, A. Tona, A. L. Plant, and J. T. Elliott, "Cell cycle dependent TN-C promoter activity determined by live cell imaging," *Cytom. Part A*, vol. 79A, no. 3, pp. 192–202, Mar. 2011.

[6] J. Chalfoun, M. Kociolek, A. Dima, M. Halter, A. Cardone, A. Peskin, P. Bajcsy, and M. Brady, "Segmenting time-lapse phase contrast images of adjacent NIH 3T3 cells.," *J. Microsc.*, vol. 249, no. 1, pp. 41–52, Jan. 2013.

[7] J. Chalfoun, M. Majurski, A. Peskin, C. Breen, and P. Bajcsy, "Empirical Gradient Threshold Technique for Automated Segmentation across Image Modalities and Cell Lines.," *J. Microsc.*, vol. 260, no. 1, pp. 86–99, 2014.

Lineage mapper: A versatile cell and particle tracker – Supplementary Note 3 – Performance Evaluation

Joe Chalfoun[5], Michael Majurski[1], Alden Dima[1], Michael Halter[2], Kiran Bhadriraju[3], and Mary Brady[1]

1 Introduction

In this document we will present the qualitative and quantitative performance evaluation for Lineage Mapper. We will describe in detail the tracking reference data generation. We will also describe the robustness of the cell tracker to the user input parameters. The robustness is measured in terms of accuracy changes with respect to the choice of the parameters. The last section describes the simulated particle tracking problem.

2 Qualitative accuracy assessment for NIH 3T3 cells

The quality of the automated segmentation and LM tracking is performed on the similarity of the computed biological outputs derived from the automated vs the manually identified cells of interest. These cells are a small subset of all cells present in the 36 FOV. These cells of interest are cells that remained in the field of view throughout a complete cell cycle, and that were well separated from other cells in the field [1]. A new cell began its cell cycle when the two daughter cells originating from a division event could be clearly distinguished. The end of the cell cycle was identified when the cell rounded and began cytokinesis for division. 257 manually segmented and tracked cells were detected over the 36 FOV whereas 344 cells were automatically detected. Figure 9 [1] shows a high similarity between the outputs generated from the automated segmentation and tracking and those generated from the manual ones. The noticeable differences arise from segmentation and background correction [1].

The NIH 3T3 datasets suggests that LM can accurately track division-to-division processes and measure cell proliferation times. This qualitative measure includes also segmentation accuracy not only tracking. The timing of mitosis differed between the manual and automated approaches because of differences in criteria for assessing mitosis. The determination in the manual analysis was based on the initial rounding

[5] Information Technology Laboratory, National Institute of Standards and Technology
2 Materials Measurement Laboratory, National Institute of Standards and Technology
3 Fischell Department of Bioengineering, University of Maryland at College Park

of the cell, whereas the automated method identified mitosis as the first frame in which two cells were clearly distinguished from one another. The manual method resulted in a time for mitosis that was on average 22 minutes or 1.46 frames sooner than the time determined by the automated method, which corresponds to approximately 3% of the cell cycle duration.

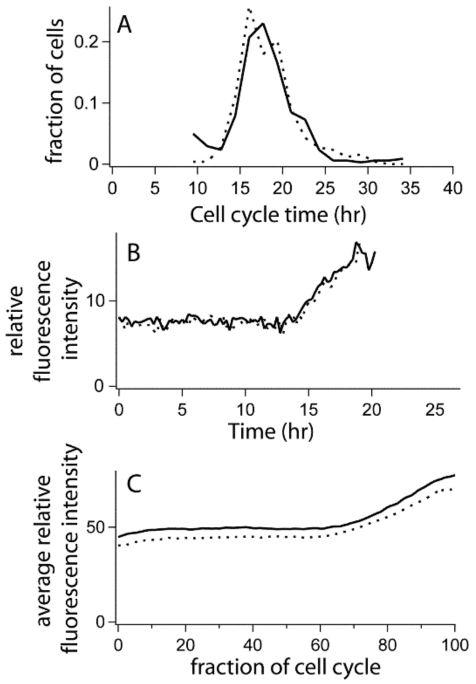

Figure 9: (A) Histogram of cell cycle times derived from manual (solid line) and automated (dashed line) segmentation and tracking. (B) Fluorescence intensity from a representative cell derived from manual (solid line; 19.25 h cell cycle time) and automated (dashed line; 20.25 h cell cycle time) segmentation and tracking versus time after division. (C) GFP intensity versus fraction of cell cycle after averaging over all manually (solid line) and automatically (dashed line) segmented and tracked cells.

3 Quantitative accuracy measurement

3.1 Reference data generation

Two datasets were segmented manually, and then manual tracking was performed on the labeled masks. Both manual segmentation and tracking data are inspected by a second expert to reduce possible human mistakes. The two datasets are: (1) NIH 3T3 cells: 238 frames acquired at 15 minute intervals, and (2) MCF10A breast epithelial sheets: 59 frames acquired at 15 minute intervals. These datasets are available for download from https://isg.nist.gov/.

3.1.1 MCF10A breast epithelial cell sheet reference dataset

Manual segmentation of breast epithelial cell sheet was performed on 59 images. An expert scientist segmented these images using ImageJ [2], contouring the cell edges using the pencil tool to set pixel values to zero. The expert worked on the raw phase images tracing all cell boundaries, leaving 1 pixel as background between cell edges in an 8 connected neighborhood. The results are shown in Figure 10. The masks are created by converting the outlines to binary in ImageJ and then filling the holes as shown in Figure 11.

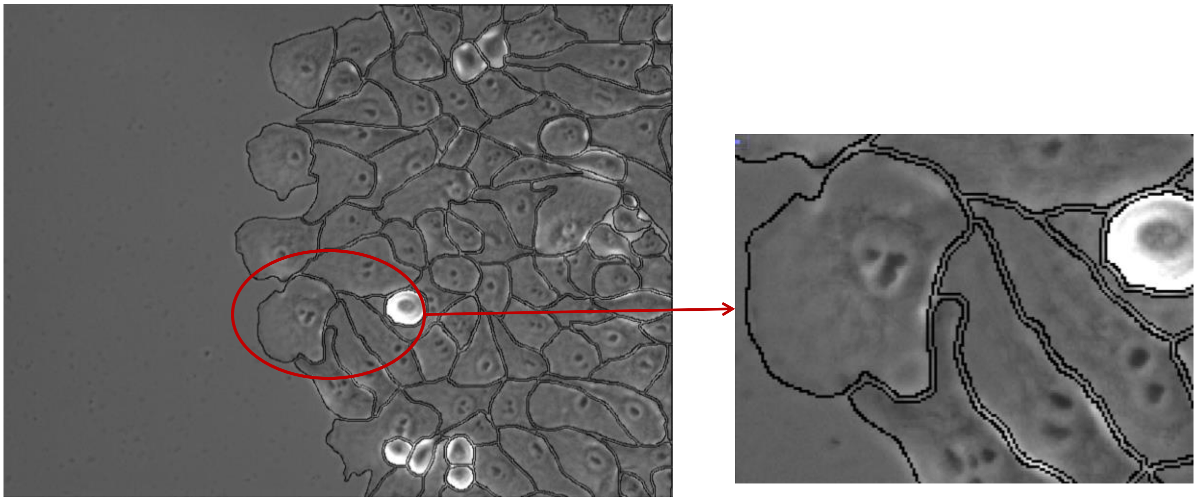

Figure 10- (Left) Manual cell separation on phase contrast image. (Right) zoomed image to show the manual boundaries that have been highlighted with the pencil tool in ImageJ.

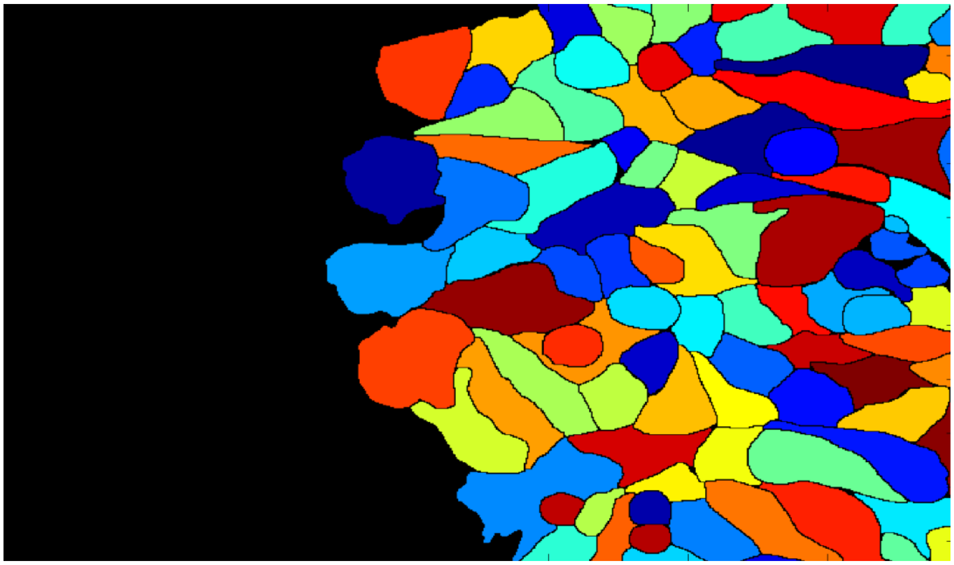

Figure 11- Labeled mask generated from the phase image after converting to binary mask, filling the holes and applying pixel connectivity algorithm. The colors are random and used only for display purposes.

3.1.2 Manual tracking

Expert scientists manually tracked previously segmented cells between consecutive frames on the NIH 3T3 dataset and the breast epithelial sheet dataset. The manual tracking results are used to generate output masks where cells are numbered with their global label from the manual tracks. The following example highlights the adopted procedure to perform manual tracking and the results are saved in an Excel spreadsheet as shown in Figure 13.

The number of rows in the Excel table is the global cell number. In Figure 13 there are four rows highlighted for this example, where both global cell numbers and local cell numbers from a particular frame are shown. For example, the cell with global cell number 6 is locally numbered 9 in the second frame, and global cell number 7 has a local number 6 in frame 2.

When a mitotic event happens, the mother cell is assigned the number 0 (the cell is dead) and the event is stored in a 2D matrix of Nx2 dimensions, where N is the total global number of cells. In the row corresponding with the division of a mother cell, the expert inputs the global numbers of its two daughter cells.

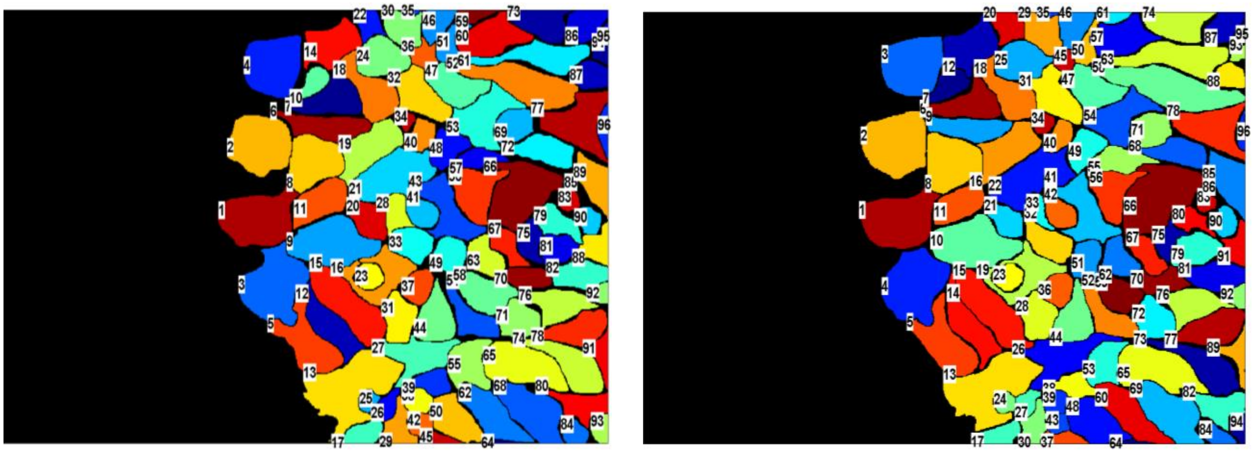

Figure 12- Two consecutive segmented images where labels are not consistent between two adjacent time points. Left is Frame 1 and Right is Frame 2.

	A	B	C	D	E	F	G	H	I	J	K	L	M	N	O	P	Q	R	S	T	U	V	W
1	1	1	1	1	1	1	1	1	1	1	1	1	1	1	1	1	1	1	1	1	1	1	1
2	2	2	2	2	2	2	2	2	2	2	2	2	2	2	2	2	2	2	2	2	2	2	2
3	3	4	3	3	3	3	3	3	3	3	3	3	3	3	3	3	3	3	3	3	3	3	3
4	4	3	4	4	4	4	4	4	4	4	4	4	4	4	4	4	4	4	4	4	4	4	4
5	5	5	6	6	6	7	6	6	6	5	5	6	6	7	6	5	6	5	5	5	6	6	5
6	6	9	7	5	5	5	7	7	5	7	6	5	5	5	5	8	5	6	6	6	5	5	6
7	7	6	5	10	9	6	5	5	7	6	7	7	7	6	7	7	7	7	7	7	8	7	7
8	8	8	8	7	8	9	10	8	8	9	8	9	9	9	9	9	9	9	9	9	9	8	9
9	9	10	9	8	7	8	8	9	9	8	9	8	8	8	8	6	8	8	8	8	7	9	8
10	10	7	12	14	11	10	9	11	11	10	11	11	11	11	11	11	11	11	11	11	11	11	11
11	11	11	10	9	10	11	11	10	10	11	10	10	10	10	10	10	10	10	10	10	10	10	10
12	12	14	13	12	14	13	13	13	13	12	14	12	13	12	12	12	12	12	12	12	12	12	12
13	13	13	11	11	12	12	12	12	12	13	12	13	12	13	13	13	13	13	13	13	13	13	13
14	14	12	14	13	13	14	14	14	14	14	13	14	14	14	14	14	14	14	14	14	14	15	14
15	15	15	15	15	15	15	15	15	15	15	15	15	15	15	15	15	15	15	15	15	15	14	15
16	16	19	17	18	20	16	19	17	19	18	17	18	17	18	18	17	18	17	17	17	18	17	17
17	17	17	18	17	17	19	18	19	20	19	19	19	19	19	19	20	19	19	19	19	19	20	20
18	18	18	19	19	18	18	17	18	17	17	18	17	18	17	21	18	17	18	18	18	17	18	18
19	19	16	16	20	16	17	16	16	16	16	16	16	16	16	16	16	16	16	16	16	16	16	16
20	20	21	20	16	19	20	20	20	18	22	21	22	21	22	22	22	22	22	22	21	22	22	23
21	21	22	21	22	21	21	21	21	21	20	20	21	20	20	20	19	21	21	21	20	20	19	19
22	22	20	23	21	22	22	22	22	23	21	22	20	22	21	23	21	20	20	20	22	21	21	21
23	23	23	24	23	23	24	24	24	24	24	24	24	24	24	25	24	25	24	25	25	24	24	25
24	24	25	25	25	25	25	25	25	25	25	25	25	25	25	17	25	24	25	24	24	25	25	24
25	25	24	22	24	24	23	23	23	22	23	23	23	23	23	24	23	23	23	23	23	23	23	22

tracking mitosis Sheet3

Ready Average: 7.222222222 Count: 9 Sum: 65 100%

Figure 13- Manual tracking spreadsheet

3.2 Simulated Particle Tracking

The simulated particle tracking described by Chenouard [3] has four scenarios: Vesicle, Microtubule, Receptor and Virus. Each scenario has three particle densities: low, medium and high and consists of 100 time-lapse images (Figure 14).

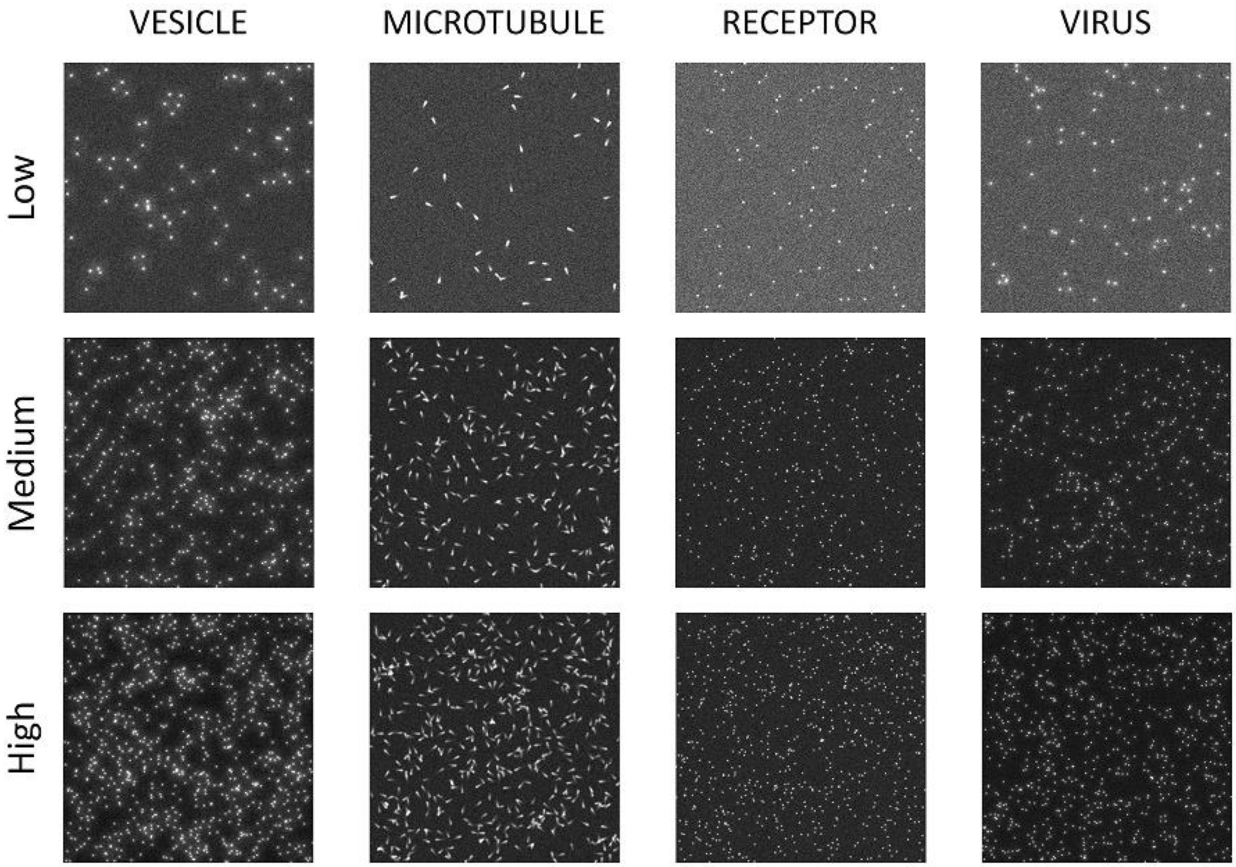

Figure 14- Simulated dataset of four scenarios (Vesicle, Microtubule, Receptor and Virus) and three densities (Low, Medium and High).

3.2.1 Segmentation

Our Cell tracker is not linked to a segmentation technique, therefore we created a simple but effective segmentation technique called PSS (Particle Simulation Segmentation) for all particle tracking simulated scenarios. We change the parameters of that technique between scenarios and densities based on the training dataset.

We separate the foreground pixels from the background ones by thresholding the image intensities with a user selected threshold. We apply pixel connectivity analysis and a user selected size threshold to reduce the noise in the segmented image leaving mainly the particles to be analyzed in the resulting mask. When particles collide with one another, we used a segmentation technique called fog bank [4] to separate them.

In the case of simulated particles, in contrast with cells, sometimes the colliding particles go on top of each other and form one slightly larger particle in size but whose intensity is much brighter than a regular particle intensity. We use this information to perform the last step of the segmentation process. We create a seed mask to detect particles that fused together by selecting the pixels that belong to the top 1 percentile in the image and we keep the objects that meet a certain user set size threshold. Those seeds are then overlaid on top of the segmented mask and each particle whose size is larger than a user set parameter will be cut into pieces equal to the number of seeds found in that particle. At the end a user set size filter is used to minimize over-segmentation. In total there are 7 user settable parameters to perform this segmentation. Table 2 gives the summary of the parameters and their respective values per scenario

and density. The open-source code of this method can be downloaded from the following webpage: https://isg.nist.gov/.

Table 2: Summary of segmentation parameters used per scenario

Parameter	VESICLES			MICROTUBULES			RECEPTORS			VIRUSES		
	Low	Med	High	Low	Med	High	Low	Med	High	Low	Med	High
Intensity Threshold	30	41	38	20	20	20	30	30	27	35	40	35
Minimum Cell Size	8	4	5	12	14	14	4	4	4	4	4	4
Minimum Seed Size	1	1	1	10	6	6	1	1	1	1	1	1
Minimum Cell Size (Fog Bank)	11	10	8	20	30	30	4	4	5	4	4	4
Brightest Pixels Seed Size	3	1	1	2	50	50	1	1	1	1	1	1
Brightest Pixels Body Size	30	25	20	90	120	120	18	15	20	15	18	20
Brightest Pixels Cell Size	8	10	11	25	14	14	5	5	5	6	4	5

3.2.2 Tracking

Our cell tracker (the Lineage Mapper) is used to track the segmented masks generated by the segmentation technique described above. The Lineage Mapper has many parameters to detect mitosis events that are disabled for particle tracking. There is a total of 6 parameters that are necessary to adjust between scenarios. A detailed explanation of these parameters can be found in the help documentation of the tool webpage at: https://isg.nist.gov/.

We found the best parameters for each scenario by running an automated optimization search over a combination of discrete parameters values that maximize the tracking accuracy on the training dataset. Table 3 gives a summary of the tracking parameters that changed between scenarios.

Table 3: Summary of tracking parameters used per scenario

Parameter	VESICLES			MICROTUBULES			RECEPTORS			VIRUSES		
	Low	Med	High	Low	Med	High	Low	Med	High	Low	Med	High
Overlap Weight	30	50	100	100	100	100	0	100	100	100	100	100
Centroids Weight	100	100	60	0	0	0	100	100	100	20	80	100
Size Weight	30	0	0	0	0	0	10	60	60	10	10	60

**Maximum Centroid Distance**	9	11	11	15	10	9	10	9	8	10	8	8
**Fusion Overlap Threshold**	60	0	0	100	60	70	100	60	60	100	50	50
**Cell Size Threshold**	9	7	8	12	14	20	5	5	5	4	4	4

3.2.3 Performance on simulated dataset

The performance of the tracking result is measured against the challenge dataset using four metrics alpha, beta, Jaccard and Jaccard Theta as presented by Chenouard [3]. We followed the same steps as highlighted by the paper and the website of the particle tracking competition. Figure 15 shows the detailed results of this analysis. There are four tables that correspond to each accuracy metric measure and twelve columns that correspond to a combination of scenario and density. Each table has 15 rows that correspond to the 15 methods being compared. All the numbers for the first 14 methods are taken from the supplementary document of Chenouard's paper and resumed in **Supplementary Table 3**. The last method (15th) is our Lineage Mapper results.

Each table is colored with 3 colors (red, green and blue) to highlight the ranking (1,2,3) respectively for each scenario and density across all method scores. Additionally, a closer look at the scores reveal only up to a second digit difference between the top positions in most cases and for all scores. Sometimes even the top 9 positions are very close in scoring like in the case of Alpha score for Vesicles low density. The last column measures the number of times a particular method ranked among the top 3 positions. This table is indicative of the robustness of a tracking technique across multiple scenarios and scoring metrics. Although the Lineage Mapper wasn't created for particle tracking, it is clear from this table that it performed well when compared against other particle tracking methods in the literature.

Scenario		VESICLES			MICROTUBULES			RECEPTORS			VIRUSES			Top 3
Density		Low	Med	High	Low	Med	High	Low	Med	High	Low	Med	High	Count
Method 1	Alpha Score	0.8881	0.7	0.5821	0.6565	0.4	0.2934	0.9237	0.4704	0.6645	0.857	0.7723	0.6647	6
Method 2		0.7892	0.6106	0.4659	0.6938	0.5644	0.4652	0.7106	0.6599	0.6007	0.8382	0.7863	0.7251	4
Method 3		0.8357	0.679	0.4891	0	0	0	0.8877	0.7878	0.695	0	0	0	2
Method 4		0	0	0	0.8464	0.6421	0.4994	0	0	0	0	0	0	3
Method 5		0.8759	0.6767	0.5334	0.8034	0.5106	0.3428	0.9068	0.7234	0.5653	0.846	0.7597	0.665	4
Method 6		0.8492	0.6121	0.4545	0.4818	0.2428	0.1555	0.8978	0.6694	0.5153	0	0	0	0
Method 7		0.6723	0.5666	0.4769	0	0	0	0.0044	0.0035	0.0036	0	0	0	0
Method 8		0.8834	0.6863	0.5273	0.6893	0.2721	0.0617	0.9098	0.7056	0.5804	0.2478	0.2535	0.2475	2
Method 9		0.8182	0.5969	0.489	0.6306	0.3595	0.2919	0.8448	0.7119	0.5933	0.6657	0.4781	0.3603	0
Method 10		0.7759	0.4267	0.2616	0	0	0	0	0	0	0	0	0	0
Method 11		0.8648	0.6346	0.4783	0.4796	0.4065	0.3356	0.9265	0.7855	0.6706	0	0	0	3
Method 12		0.8608	0.6052	0.367	0.5141	0.237	0.1242	0.8935	0.5975	0.4196	0.7173	0.5518	0.385	0
Method 13		0.7337	0.5153	0.3842	0.4305	0.2459	0.1723	0.7898	0.5995	0.4721	0.5519	0.342	0.2213	0
Method 14		0.7077	0.4915	0.3739	0.4089	0.2516	0.1737	0.6141	0.5825	0.4858	0.1221	0.1658	0.1344	0
Method 15		0.8921	0.6983	0.5762	0.7778	0.5181	0.3852	0.916	0.7914	0.7011	0.9321	0.8295	0.7303	12
Method 1	Beta Score	0.851	0.6193	0.4942	0.6214	0.3259	0.2308	0.9045	0.4022	0.5949	0.8463	0.7365	0.6123	6
Method 2		0.763	0.5474	0.3953	0.6861	0.5347	0.4274	0.6097	0.5662	0.5131	0.7987	0.7383	0.6729	4
Method 3		0.8154	0.6168	0.3832	0	0	0	0.8734	0.7539	0.6497	0	0	0	2
Method 4		0	0	0	0.8335	0.6	0.4482	0	0	0	0	0	0	3
Method 5		0.8492	0.6048	0.4526	0.7872	0.4501	0.2794	0.8922	0.6726	0.4868	0.8374	0.7265	0.6171	5
Method 6		0.8132	0.5372	0.3782	0.4388	0.192	0.1182	0.8749	0.6116	0.4511	0	0	0	0
Method 7		0.6639	0.5238	0.4163	0	0	0	0.0022	0.0018	0.0018	0	0	0	0
Method 8		0.8445	0.6224	0.4618	0.6401	0.2176	0.044	0.8742	0.6704	0.5232	0.1776	0.1792	0.1761	2
Method 9		0.7998	0.4985	0.3978	0.5972	0.2941	0.2387	0.828	0.6565	0.5227	0.6313	0.4175	0.3074	0
Method 10		0.6341	0.2963	0.1967	0	0	0	0	0	0	0	0	0	0
Method 11		0.8396	0.5633	0.3951	0.4566	0.3515	0.2818	0.9078	0.7443	0.6108	0	0	0	3
Method 12		0.8427	0.54	0.2836	0.4657	0.1694	0.0802	0.8742	0.5163	0.3277	0.6953	0.4905	0.3017	0
Method 13		0.6929	0.4499	0.3268	0.3975	0.1976	0.1362	0.7652	0.542	0.4105	0.4955	0.3013	0.2007	0
Method 14		0.6697	0.4197	0.3001	0.3761	0.2116	0.144	0.5226	0.5139	0.4241	0.0904	0.1145	0.0923	0
Method 15		0.8716	0.62	0.481	0.7473	0.4401	0.3117	0.9053	0.7458	0.6262	0.9204	0.7896	0.6695	11
Method 1	Jaccard Score	0.8717	0.6501	0.5258	0.8366	0.4482	0.3221	0.9175	0.4388	0.6228	0.9608	0.8393	0.7025	3
Method 2		0.8278	0.6059	0.4575	0.954	0.763	0.6274	0.6701	0.6269	0.5765	0.8904	0.8271	0.7566	5
Method 3		0.8835	0.6726	0.4325	0	0	0	0.9465	0.8254	0.7169	0	0	0	5
Method 4		0	0	0	0.9336	0.6943	0.5386	0	0	0	0	0	0	3
Method 5		0.8823	0.646	0.5035	0.8935	0.5581	0.3777	0.916	0.7077	0.537	0.9527	0.8252	0.7179	3
Method 6		0.8344	0.5595	0.4055	0.7552	0.3448	0.2142	0.8925	0.6322	0.4782	0	0	0	0
Method 7		0.9208	0.7244	0.5875	0	0	0	0.0052	0.0045	0.0044	0	0	0	3
Method 8		0.8557	0.6396	0.4944	0.6878	0.2767	0.0748	0.887	0.7093	0.5638	0.3073	0.3099	0.3013	0
Method 9		0.8751	0.5534	0.4549	0.7855	0.3984	0.3223	0.8935	0.7208	0.5818	0.845	0.5847	0.4648	0
Method 10		0.6686	0.346	0.2476	0	0	0	0	0	0	0	0	0	0
Method 11		0.8558	0.5865	0.4246	0.8598	0.5696	0.4369	0.925	0.7676	0.6429	0	0	0	5
Method 12		0.8694	0.5725	0.3277	0.6776	0.2734	0.1373	0.8978	0.5634	0.3809	0.8695	0.6289	0.4152	0
Method 13		0.8128	0.5424	0.4087	0.7785	0.3798	0.2588	0.8947	0.6502	0.5074	0.7028	0.4089	0.2796	0
Method 14		0.7866	0.5044	0.3702	0.7704	0.4065	0.294	0.6157	0.6133	0.5187	0.2171	0.2292	0.1885	0
Method 15		0.9116	0.6586	0.5256	0.8694	0.519	0.3868	0.9535	0.7799	0.6605	0.9669	0.8258	0.7111	9
Method 1	Jaccard Theta Score	0.9068	0.7583	0.683	0.9019	0.6458	0.5824	0.9479	0.5722	0.7643	0.9782	0.8909	0.7941	2
Method 2		0.9174	0.7752	0.6648	0.9588	0.8469	0.7542	0.7224	0.7138	0.6885	0.9211	0.8722	0.8435	4
Method 3		0.9434	0.8095	0.5671	0	0	0	0.957	0.8957	0.8242	0	0	0	4
Method 4		0	0	0	0.9507	0.8026	0.7067	0	0	0	0	0	0	3
Method 5		0.9151	0.7827	0.698	0.9385	0.7386	0.613	0.9489	0.8323	0.7203	0.9698	0.876	0.806	6
Method 6		0.9083	0.7382	0.6218	0.8451	0.5731	0.4546	0.919	0.7617	0.6349	0	0	0	0
Method 7		0.9767	0.8776	0.791	0	0	0	0.0111	0.0167	0.0149	0	0	0	3
Method 8		0.9106	0.7883	0.65	0.7557	0.3898	0.127	0.9341	0.8331	0.7229	0.4087	0.3862	0.3754	0
Method 9		0.9158	0.6225	0.5942	0.8612	0.6355	0.5757	0.9363	0.8067	0.697	0.9013	0.6985	0.6249	0
Method 10		0.7014	0.5067	0.4635	0	0	0	0	0	0	0	0	0	0
Method 11		0.9286	0.7865	0.6657	0.8961	0.7044	0.6174	0.9489	0.8579	0.7749	0	0	0	3
Method 12		0.9452	0.7464	0.5155	0.8262	0.4715	0.319	0.9226	0.6766	0.515	0.8845	0.701	0.532	1
Method 13		0.8662	0.6768	0.5899	0.8502	0.5913	0.5159	0.9293	0.7544	0.6631	0.776	0.5864	0.4952	0
Method 14		0.8487	0.6295	0.5086	0.8467	0.6142	0.5609	0.658	0.7186	0.6723	0.316	0.3302	0.2981	0
Method 15		0.9496	0.7897	0.6831	0.9414	0.6968	0.6171	0.9677	0.8897	0.7922	0.9799	0.8902	0.8073	10

Rank: 1 (red), 2 (green), 3 (blue)

Figure 15: Summary of the accuracy results of the simulated dataset compared to 14 other trackers. The accuracy results as measured by each metric are displayed. The colors are the rankings per scenario/density among all methods: red is the highest, green is the second and blue the third. The last column measures the number of times a particular method ranked among the top 3 positions.

4 Robustness to user input

In order to test the robustness of the cell tracking technique against parametric changes, we performed a sensitivity analysis over the NIH 3T3 data, for which manual tracking is available.

The sensitivity analysis is performed with respect to the 3 weights of the cost function: overlap, centroid distance and size weights. Each weight is assigned independently. The goal is to study the performance change of the cell tracking as a function of parametric weight. Each weight can take the following percent values [20, 40, 60, 80, 100], for a total of $5^3 = 125$ different combinations. We run the automated tracking and compute the accuracy of tracking and mitotic detection for each combination. The result is plotted in Figure 16 in 4D, where the fourth dimension is the accuracy value given in %. This plot shows that the cell tracker performance is very robust against variations of weights. Only 34 combinations out 125 yielded accuracy less than 90 %. One can notice that the performance is worse when the overlap weight is minimal (at 20 %) and the size weight is maximal (=100 %). On the other hand, tracking performance peaks when the overlap weight is above 60 %. This is due to the fact that in this example overlap is very important.

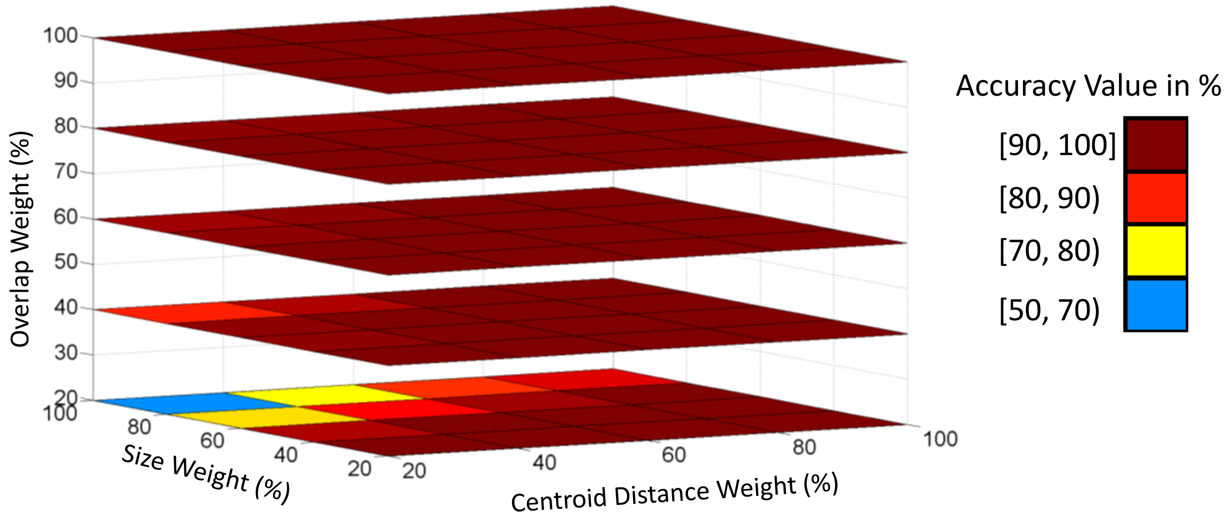

Figure 16- Overall sensitivity analysis. The (x,y,z) are respectively the weight of cell size, centroid distance and the overlap. The accuracy (the fourth dimension) is color-coded: light blue corresponds to the cell tracker achieving an accuracy of 55 % with the weight combination of (100,20,20) and dark red corresponds to an accuracy higher than 90 % with the maximum equals to 94.42 % with a weight combination of (a,b,100) with $\boldsymbol{a}, \boldsymbol{b} \in [\mathbf{20}, \mathbf{100}]$

5 References

[1] M. Halter, D. R. Sisan, J. Chalfoun, B. L. Stottrup, A. Cardone, A. a. Dima, A. Tona, A. L. Plant, and J. T. Elliott, "Cell cycle dependent TN-C promoter activity determined by live cell imaging," *Cytom. Part A*, vol. 79A, no. 3, pp. 192–202, Mar. 2011.

[2] J. Schindelin, I. Arganda-Carreras, E. Frise, V. Kaynig, M. Longair, T. Pietzsch, S. Preibisch, C. Rueden, S. Saalfeld, B. Schmid, J.-Y. Tinevez, D. J. White, V. Hartenstein, K. Eliceiri, P. Tomancak, and A. Cardona, "Fiji: an open-source platform for biological-image analysis.," *Nat. Methods*, vol. 9, no. 7, pp. 676–82, Jul. 2012.

[3] N. Chenouard, I. Smal, F. de Chaumont, M. Maška, I. F. Sbalzarini, Y. Gong, J. Cardinale, C.

Carthel, S. Coraluppi, M. Winter, A. R. Cohen, W. J. Godinez, K. Rohr, Y. Kalaidzidis, L. Liang, J. Duncan, H. Shen, Y. Xu, K. E. G. Magnusson, J. Jaldén, H. M. Blau, P. Paul-Gilloteaux, P. Roudot, C. Kervrann, F. Waharte, J.-Y. Tinevez, S. L. Shorte, J. Willemse, K. Celler, G. P. van Wezel, H.-W. Dan, Y.-S. Tsai, C. Ortiz de Solórzano, J.-C. Olivo-Marin, and E. Meijering, “Objective comparison of particle tracking methods.,” *Nat. Methods*, vol. 11, no. 3, pp. 281–9, Mar. 2014.

[4] J. Chalfoun, M. Majurski, A. Dima, C. Stuelten, and A. Peskin, “FogBank : A Single Cell Segmentation across Multiple Cell Lines and Image Modalities,” *BMC Bioinformatics*, no. submitted for review, 2014.
